# Evaluation of the Genetic Basis of Familial Aggregation of Pacemaker Implantation by a Large Next Generation Sequencing Panel

**DOI:** 10.1371/journal.pone.0143588

**Published:** 2015-12-04

**Authors:** Patrícia B. S. Celestino-Soper, Anisiia Doytchinova, Hillel A. Steiner, Andrea Uradu, Ty C. Lynnes, William J. Groh, John M. Miller, Hai Lin, Hongyu Gao, Zhiping Wang, Yunlong Liu, Peng-Sheng Chen, Matteo Vatta

**Affiliations:** 1 Department of Medical and Molecular Genetics, Indiana University School of Medicine, Indianapolis, IN, United States of America; 2 Krannert Institute of Cardiology, Division of Cardiology, Department of Medicine, Indiana University School of Medicine, Indianapolis, IN, United States of America; 3 Center for Computational Biology and Bioinformatics, Indiana University Purdue University Indianapolis, Indianapolis, IN, United States of America; 4 Baruch Padeh Medical Center, Poriya MP Lower Galilee, Israel; 5 Faculty of Medicine in the Galilee, Bar-Ilan University, Safed, Israel; University of Milan, ITALY

## Abstract

**Background:**

The etiology of conduction disturbances necessitating permanent pacemaker (PPM) implantation is often unknown, although familial aggregation of PPM (faPPM) suggests a possible genetic basis. We developed a pan-cardiovascular next generation sequencing (NGS) panel to genetically characterize a selected cohort of faPPM.

**Materials and Methods:**

We designed and validated a custom NGS panel targeting the coding and splicing regions of 246 genes with involvement in cardiac pathogenicity. We enrolled 112 PPM patients and selected nine (8%) with faPPM to be analyzed by NGS.

**Results:**

Our NGS panel covers 95% of the intended target with an average of 229x read depth at a minimum of 15-fold depth, reaching a SNP true positive rate of 98%. The faPPM patients presented with isolated cardiac conduction disease (ICCD) or sick sinus syndrome (SSS) without overt structural heart disease or identifiable secondary etiology. Three patients (33.3%) had heterozygous deleterious variants previously reported in autosomal dominant cardiac diseases including CCD: *LDB3* (p.D117N) and *TRPM4* (p.G844D) variants in patient 4; *TRPM4* (p.G844D) and *ABCC9* (p.V734I) variants in patient 6; and *SCN5A* (p.T220I) and *APOB* (p.R3527Q) variants in patient 7.

**Conclusion:**

FaPPM occurred in 8% of our PPM clinic population. The employment of massive parallel sequencing for a large selected panel of cardiovascular genes identified a high percentage (33.3%) of the faPPM patients with deleterious variants previously reported in autosomal dominant cardiac diseases, suggesting that genetic variants may play a role in faPPM.

## Introduction

Cardiac conduction disease (CCD) and sick sinus syndrome (SSS) are common indications for pacemaker implantation [1–4]. According to the current guidelines, genetic testing may be considered for patients with isolated CCD (ICCD) or CCD with concomitant congenital heart disease, especially with documented remarkable family history of cardiac conduction disease. Genetic testing for ICCD remains a Class IIb recommendation, as variants in *SCN5A*, which cause the majority of familial cardiac conduction disease cases, account for only about 5% of the disease burden [5]. While other genes including *SCN1B*, *TRPM4*, and *HCN4* have been associated with idiopathic sinus node dysfunctions (SNDs) [5], it is unclear if more comprehensive testing would yield additional useful results.

The development of next generation sequencing (NGS) has brought an economical and efficient way to detect variants in thousands of target regions. Several targeting approaches have been developed for NGS including whole genome sequencing (WGS), whole exome sequencing (WES), and disease-targeted panels. WGS and WES methods are better suited for very rare disorders for which no other clinical genetic tests are available. In comparison, the gene panels can be designed with a specific clinical phenotype/s or disease/s in mind, allowing a more targeted approach for result interpretation and subsequent patient management recommendations. Unlike WGS and WES, gene panels also permit a better sequencing coverage of targeted regions, more accurate and reliable analyses, and overall lower cost [6, 7]. Here we describe the application of a custom NGS targeted enrichment method to implement a research panel for the study of selected patients with familial aggregation of permanent pacemaker implantation (faPPM). Currently, there is limited knowledge about the role of genetic predisposition to faPPM. Here we present the findings resulting from a comprehensive genetic approach using a large panel including genes associated with cardiomyopathies, arrhythmias, congenital heart defects, aortopathy, connective tissue disorders, Noonan spectrum disorders, pulmonary arterial hypertension, metabolic disorders with cardiac presentation, and lipid disorders.

## Materials, Methods, and Patient Selection Methodology

This study protocol was approved by the Institutional Review Board of the Indiana University School of Medicine. All participants have provided written informed consent to participate in this study. A total of 112 patients with permanent pacemaker (PPM) implantation were recruited from a pacemaker clinic. Among them, nine (8%) presented with ICCD or SSS without structural heart disease and had at least one first degree relative (FDR) with PPM, thus described to have faPPM and their DNAs were used for NGS analyses. Table 1 summarizes the characteristics of all recruited patients including those submitted for genetic analysis. Exclusion criteria for patients not submitted for genetic analysis are listed in Table A in S1 File. A description of each patient at the time of their study enrollment and a summary of their clinical course as available in the medical records is described in S1 File (patient description section).

**Table 1:** Patient characteristics.

	All patients	No family history‡	Positive family history‡	Family history + ICCD/SSS‡	Genotype positive‡	Genotype negative‡
N (%)	112	88(78.6%)	24(21.4%)	9(8%)	3(33.3%) *	6(66.7%) *
Male	59(52.7%)	47(53.4%)	12(50%)	5(55.6%)	2(66.7%)	3(50%)
Female	53(47.3%)	41(46.6%)	12(50%)	4(44.4%)	1(33.3%)	3(50%)
Age (mean±SD)	69.6±14.8	69.9±15.5	68.8±11.9	67.7±9.9	68.7±11.2	67.2±10.2
Age (range)	28–94	28–94	39–87	48–81	59–81	48–77
Implant age (mean±SD)	61.2±16.9	62.1±16.6	58±17.9	53.7±17.7	49.3±22.5	55.8±16.7
Implant age (range)	11–88	11–88	15–83	24–71	24–68	24–71
Caucasian	97(86.6%)	77(87.5%)	20(83.3%)	7(77.8%)	3(100%)	4(66.7%)
African American	14(12.5%)	10(11.4%)	4(16.7%)	2(22.2%)	0(0%)	2(33.3%)
Hispanic	1(0.9%)	1(1.1%)	0(0%)	0(0%)	0(0%)	0(0%)
Dependency†	27(24.3%)	20(23%)	7(29.2%)	3(33.3%)	2(66.7%)	1(16.7%)
Conduction disturbance	32(28.6%)	27(30.7%)	5(20.8%)	3(33.3%)	1(33.3%)	2(33.3%)
Complete AVB	39(34.8%)	30(34.1%)	9(37.5%)	4(44.4%)	2(66.7%)	2(33.3%)
Sick sinus syndrome	41(36.6%)	31(35.2%)	10(41.7%)	2(22.2%)	0(0%)	2(33.3%)
Atrial fibrillation†	43(38.7%)	35(40.2%)	8(33.3%)	0(0%)	0(0%)	0(0%)

**ICCD** = isolated cardiac conduction disease; **SSS** = sick sinus syndrome in absence of structural heart disease.

*Percentage as compared to patients with ICCD and positive family history.

^†^Determined in 111 patients, one patient had missing data.

^‡^Fisher’s test was performed to obtain the p-value for “No FHx” vs. “POS FHx”, “POS FHx” vs.”POS FHx +ICCD/SSS”, and “GT POS” vs. “GT NEG” columns for the following items (rows in table): “Caucasian”, “African American”, “Hispanic”, “Dependency”, “Conduction disturbance”, “Complete AVB”, “Sick sinus syndrome”, and “Atrial fibrillation”. None of the p-values obtained reached significance (that is, none were below 0.05); note that n is small for all tested categories. For the remaining items—rows “N (%)”, “Male”, “Female”, “Age (mean±SD)”, “Age (range)”, “Implant age (mean±SD)”, and “Implant age (range)” -, statistical analyses was not applicable/not available due to insufficient categories for the performance of a Fisher’s test.

Post-bioinformatics filtering of variants found in the 9 pacemaker individuals started with categorization of variants as either present or absent in the Human Gene Mutation Database (HGMD) [8]. Table H in S1 File summarizes the initial classification of HGMD variants, which was followed by re-classification, if needed, after careful literature analyses. The following literature evidence was used to re-classify the HGMD variants:

1. Affects protein function
  - Affects function in isolation: truncating variant in gene where loss of function is a known mechanism of disease; or variant found to be disease causing from linkage studies or co-segregation studies in large families. In all cases, there must be sufficient functional data to support a deleterious effect of the variant and the variant must have frequency of less than or equal to 5% in all combined populations in both the Exome Sequencing Project (ESP) and the 1000 Genomes project (1000G) (hg19 data from ANNOVAR [9]). These variants were confirmed by Sanger sequencing.
  - May or may not affect function in isolation: *de novo* variant in a patient with the disease and unaffected parents; or co-segregation studies in small to mid-sized families. In all cases, there must be sufficient functional data to support a deleterious effect of the variant and the variant must have frequency of less than or equal to 5% in all combined populations in both the ESP and the 1000G.
  - Affects function as a modifier: same requirements as the two categories above, but ESP or 1000G frequency must be above 5% and smaller than or equal to 50%.
2. Variant of Unknown Significance (VUS): literature support for disease associated variant (without functional data) or literature support for deleterious effect of the variant (without disease association). In all cases, the variant must have frequency of less than or equal to 5% in all combined populations in both the ESP and the 1000G.
3. Likely does not affect function/does not affect function: literature support for lack of pathogenicity; or frequency above 50% in ESP or 1000G for variant that would otherwise be classified in category 1.3; or frequency above 5% in ESP or 1000G for variant that would otherwise be classified in category 2.

Variants that were not found in the HGMD database were classified as VUSs. To filter out variants that would likely not affect protein function, we retained splicing (within 2 bp of splicing junction) and exonic variants (except synonymous) that had a frequency <5% in all combined populations in both the ESP and the 1000G (hg19 data from ANNOVAR [9]), and that had a damage score of 4 or above (see Table G in S1 File). The selected variants above were then subdivided into either being recurrent (found in 2 or more pacemaker patients) or non-recurrent. Recurrent variants that were found to be false positive (FP) in the Coriell samples used for validation were eliminated. Variants that were located within *TNXB* and *ADAMTSL2* and within regions of known segmental duplications were excluded for potentially being FP variants. Frame-shift, premature stop, and splicing VUSs were confirmed by Sanger. For both HGMD and nonHGMD variants, a cut-off for the Minor Allele Frequency (MAF) of 5% was used because in addition to rare and highly penetrant variants, previous reports have implicated common functional genetic variants leading to arrhythmia susceptibility or variable ECG traits including SND. Moreover, it is well known that genetic arrhythmias, although mostly of monogenic and Mendelian basis, may be caused by multiple deleterious DNA variants as it happens in up to 8.5% of subjects with long QT syndrome (LQTS), usually leading to more severe phenotypes [10]. In summary, a 5% MAF cutoff was used to capture rare and highly penetrant deleterious variants, which alone could be responsible for the SND clinical presentation, along with well known or potential more common susceptibility variants, which could play a significant role in the development of SND. Moreover, the recently released “Standards and guidelines for the interpretation of sequence variants” issued by consensus recommendation of the American College of Medical Genetics and Genomics and the Association for Molecular Pathology also suggested that allele frequency >5% in Exome Sequencing Project, 1000 Genomes Project, or Exome Aggregation Consortium for a rare Mendelian disorder may be used as stand-alone evidence of benign impact [11]. Provided that PPM occurs at a relative high frequency in the population, that genetic variants may not be highly penetrant, and that multiple deleterious variants may be necessary to cause a clinically relevant presentation, our cut-off of <5% appears to be reasonable.

Information regarding panel design, library preparation, sequencing procedures, bioinformatics pipeline and data analysis, panel validation, additional post-bioinformatics variant filtering procedures, Sanger sequencing for confirmation of variants, and analytic performance assessment are found in S1 File (materials and methods section). Due to the nature of the study, we did not have the permission to contact the affected relatives of the index cases to obtain in-depth clinical information and to enroll them in the genetic study. Therefore, as also explained in the “Limitation Study” section, co-segregation analysis of the putative pathogenic variants was not possible.

## Results

Nine faPPM patients were studied using an Agilent HaloPlex custom pan-cardiovascular disease targeted panel for NGS. The NGS panel was designed to detect variants in the splicing and coding regions of 246 genes associated with cardiovascular disorders including cardiomyopathies, arrhythmias, congenital heart defects, aortopathy, connective tissue disorders, Noonan spectrum disorders, pulmonary arterial hypertension, metabolic disorders that afflict the heart, and lipid disorders. Commercially available DNA samples from seven genotype-known individuals from Coriell were sequenced to estimate target coverage and to optimize and validate the panel. Detailed results regarding coverage, sequence depth, inter and intra-run variability using Coriell and pacemaker samples, and the HaloPlex SNP performance for Coriell sequencing runs are found in S1 File (results section). Overall, we obtained consistent coverage within and between MiSeq sequence runs. The precision of the HaloPlex custom panel sequence runs and the variability in sequence depth among runs showed acceptable variability. Validation experiments performed with Coriell samples showed that our custom NGS platform and bioinformatics analysis pipeline can produce data with less than 8% false positive (FP) calls (range of 2.33 to 7.32) and less than 5% false negative (FN) calls (range of 0.63 to 4.23) for exonic and splicing regions of all 246 cardiovascular genes with a minimum of 15x sequencing depth (95% of targets, see Table B in S1 File) after removal of regions of segmental duplications. The overall pacemaker patient target region average sequence depth and the exonic and splicing only (focus regions of our analyses) sequence depth were calculated for our experiments and resulted in an average of 194x and 200x, respectively (Table B in S1 File). Finally, we observed that at a minimum of 15x sequence depth, an average of 95% of target regions were covered with a mean read depth of 205x for pacemaker patients (Table B in S1 File).

### Distribution of Variants in ICCD or SSS

Table A in S2 File summarizes the distribution of variants found in each pacemaker patient. An average of 2,344 variants was obtained per patient, with 83.2% of variants mapping to non-exonic and non-splicing positions. Although not intentionally targeted, these intronic, untranslated region (UTR), and intergenic variants come from the library preparation process, which, as expected, does not limit the capture and enrichment process to the exact coordinates of the exons and splicing regions. Additionally, the large occurrence of non-exonic/non-splicing variants is expected given their tendency to be less conserved, supporting the observation that only 0.3% of non-exonic and/or non-splicing variants were documented in the Human Gene Mutation Database (HGMD). This supports the removal of non-HGMD (not present in the HGMD database) non-exonic/non-splicing variants, as described in S1 File (methods section). Likewise, an average of 392 exonic variants was obtained per patient, with nearly 60% of exonic variants being synonymous (silent) and almost 39% of exonic variants being non-synonymous (missense) (Table A in S2 File). Additionally, it was observed that only 2.9% of synonymous variants were found in HGMD, supporting that elimination of non-HGMD synonymous variants, as described in S1 File (methods section), is an acceptable step in the process of finding disease causing variants. A limited number of splicing, stop-gain, stop-loss, frameshift and non-frameshift variants were found in the pacemaker patients.

### Classification of Variants in Genotype Unknown ICCD or SSS

Non-HGMD variants were filtered as described in S1 File (materials and methods section) to select a group of variants of unknown significance (VUSs) with potential involvement in the patients’ phenotype (Table C in S1 File). Although these variants have not been previously reported, they are rare in the general population and had a high prediction score of potentially affecting protein function. A minimum of 2 and maximum of 10 non-HGMD VUSs were found in all pacemaker patients (Table D in S1 File). It was hypothesized that genes associated with arrhythmias (Table B in S2 File) may be strong candidates in our pacemaker patients, given their ICCD or SSS presentation and a family history of pacemaker implantation. Eight of our 9 pacemaker patients had at least one VUS in an arrhythmia associated gene (Tables C and D in S1 File).

HGMD documented variants were categorized as described in S1 File (materials and methods section) to come up with a well annotated group of variants. After careful literature review, approximately 33% of HGMD variants were re-classified compared to their original classification (see Table D in S1 File). The final list of re-categorized HGMD VUSs is presented in Table C in S1 File, while the final list of re-categorized HGMD variants that affect protein function in isolation (category 1.1), variants that may or may not affect function in isolation (category 1.2), or variants that affect function as modifiers (category 1.3) is presented in Table E in S1 File. Three out of 9 patients had previously reported pathogenic variants that affect function in isolation (category 1.1); however, 3 of those variants in 2 patients are known to be pathogenic in a homozygous state, whereas the patients were heterozygous (Tables D and E in S1 File). Five out of 9 pacemaker patients had previously reported pathogenic variants that may or may not affect function in isolation (category 1.2), and two of our patients had two of these variants (Tables D and E in S1 File). Overall, patients that had previously reported deleterious variants that could explain their phenotype include those that had at least one variant in category 1.1 or at least two variants in category 1.2. This aggregation resulted in three out of nine of the pacemaker patients (33.3%) that had deleterious variants that could explain their phenotype (patients 4, 6, and 7). Patient 4 had a category 1.1 variant in the *LDB3* gene—heterozygous c.349G>A (p.D117N), NM_001080116, rs121908338 (frequency of approximately 1% in the 1000G database and 0.7% in the ESP database), and a category 1.2 variant in the arrhythmia associated gene *TRPM4* –heterozygous c.2531G>A (p.G844D), NM_017636, rs200038418 (frequency of approximately 0.05% in the 1000G database and 0.1% in the ESP database). Patient 6 also had the heterozygous *TRPM4* above, in addition to a second category 1.2 variant in the arrhythmia associated gene *ABCC9*—heterozygous c.2200G>A (p.V734I), NM_005691, rs61688134 (frequency of approximately 1% in the 1000G database and 0.9% in the ESP database and 0.7% in the ExAC database). Patient 7 had a category 1.2 variant in the *APOB* gene—heterozygous c.10580G>A (p.R3527Q), NM_000384, rs5742904 (not found in the 1000G database and 0.07% frequency in the ESP database), and a category 1.2 variant in the arrhythmia associated gene *SCN5A*—heterozygous c.659C>T (p.T220I), NM_198056, rs45620037 (frequency of approximately 0.05% in the 1000G database and 0.03% in the ESP database).

A comparison of the characteristics of genotype positive and genotype negative patients is also included in Table 1. Notably, all genotype positive patients were Caucasian. While the average age at study recruitment was similar among all groups of patients studied, the genotype positive patients on average had their PPM implanted at an earlier age compared to all patients with pacemakers (49.3 ± 22.5 vs 55.8 ± 16.7 years) (Table 1). No statistically significant differences were found using Fisher’s test between the no family history and the positive family history subjects or between the positive family history and the positive family history + ICCD/SSS subjects for the categories analyzed (ethnicities, dependency, conduction disturbance, complete AVB, sick sinus syndrome, and atrial fibrillation). None of the genotype positive patients had co-morbidities at the time of PPM implantation. A list of major co-morbidities in patients submitted for genetic analysis is listed in Table F. The nine patients with ICCD or SSS without structural heart disease had clinical data available for 18.1 ± 9.7 years after device implantation. Six of them did not develop symptomatic congestive heart failure or coronary artery disease, although echocardiographic abnormalities such as left ventricular hypertrophy or diastolic dysfunction were common. There was one patient in the genotype positive group and two in the genotype negative group who developed coronary artery disease during follow-up. Detailed clinical follow-up data of all patients submitted for genetic analysis is listed in Table 2. We were able to locate ECGs from 5 of the 9 patients studied (see S1 Fig). Among them, the ECGs of patients 2 and 4 showed complete heart block. ECG of patient 6 showed ventricularly paced rhythm (ECG prior to pacemaker implant was not available). ECG of patient 9 showed 2:1 (most likely Mobitz II) AV block. The ECG of patient 8 showed profound sinus bradycardia, junctional rhythm with AV dissociation, consistent with sick sinus syndrome. The ECGs of the remaining 4 patients were not available. Out of the two patients with congenital heart block in the absence of congenital heart disease, one was genotype positive for the *LDB3* and *TRPM4* variants (patient 4) and the other one was negative. In the patient who was genotype negative (patient 2), while the etiology of the complete heart block was listed as unknown, suspected etiology included both congenital heart block and history of scarlet fever at age 1. Given that no deleterious variants were identified in her case, in retrospect, her conduction disorder could be due to her scarlet fever in childhood.

**Table 2:** Follow up after pacemaker insertion in patients who had ICCD or SSS without structural heart disease.

Patient number	PPP Indication	Mutation^‡^	Follow up (years)	Outcome
1	CHB	None	4.4	No evidence of heart failure symptoms* or coronary disease; developed recurrent complications from COPD and alcohol abuse
2	Congenital CHB vs CHB due to scarlet fever†	None	29	Developed coronary artery disease and heart failure 20 years after PPM insertion
3	SSS	None	12	No evidence of heart failure symptoms* and coronary artery disease; developed lupus nephritis
4	CHB	*LDB3* (1.1); *TRPM4* (1.2)	15	No evidence of heart failure symptoms* or coronary artery disease
5	Mobitz II ABV	None	8.9	Developed coronary artery disease 3 years after PPM insertion and recurrent strokes
6	2:1 AVB	*TRPM4* (1.2); *ABCC9* (1.2)	17.3	No evidence of heart failure* symptoms or coronary artery disease
7	Congenital CHB	*APOB* (1.2); *SCN5A* (1.2)	35.8	Developed coronary artery disease and atrial fibrillation >20 years after PPM insertion
8	SSS	None	20.7	No evidence of heart failure symptoms or coronary artery disease; developed atrial fibrillation
9	Mobitz II AVB	None	20	No evidence of heart failure symptoms or coronary artery disease; developed dementia
Average			**18.1±9.8**	

*Excluding stage 1 diastolic dysfunction and/or left ventricular hypertrophy on echocardiogram.

^†^In this patient the reason for the complete heart block was unknown, it was postulated that it was either due to congenital complete heart block or history of scarlet fever at one year of age.

^‡^Patients that had pathogenic mutations that could explain their phenotype include those that had at least one mutation in category 1.1 or at least 2 mutations in category 1.2. Here the gene (s) in categories 1.1 and 1.2 are listed per patient.

**AVB** = atrioventricular block; **CHB** = complete heart block; **COPD** = chronic obstructive pulmonary disease; **PPM** = permanent pacemaker; **SSS** = sick sinus syndrome.

## Discussion

Conduction defects represent a major cause of morbidity and mortality worldwide. Pacemaker implantation or a dual device (pacemaker and ICD) implantation remains an appropriate option in subjects presenting with SND symptoms. However, in this study, we have observed that familial aggregation of PPM (faPPM) occurs in 9/112 (8%) patients evaluated in our clinic, thus representing a significant proportion of all individuals requiring a PPM. The occurrence of faPPM suggests a possible genetic predisposition to the development of SND, thus making those subjects candidates for genetic screening. Here, we show that a comprehensive genetic approach using a targeted pan-cardiovascular 246 genes panel could identify at least one previously reported pathogenic category 1.1 or at least two category 1.2 variants as the most likely explanation of their clinical phenotypes as described below.

The *LDB3* gene mapping to chromosome 10q23.2 codes for the protein ZASP, which is highly expressed in the heart and skeletal muscle and is a crucial component of the sarcomeric Z-disks in binding critical sarcomeric proteins including alpha actinin-2, protein kinase C, and myozenin family proteins (OMIM 605906) [12, 13]. Variants in *LDB3* have been associated with dilated cardiomyopathy (DCM), left ventricular noncompaction (LVNC), and myofibrillar myopathy. The heterozygous *LDB3* variant seen in pacemaker patient 4, p.D117N, has been previously seen in heterozygosity in two unrelated individuals with DCM/LVNC and conduction defects [14]. Previous functional analyses in HEK293 and neonatal rat cardiomyocytes demonstrated that ZASP interacts with the *SCN5A*-coded sodium channel Na_v_1.5, and affects the channel voltage-dependent activation and inactivation, leading to loss of function of Na_v_1.5, consistent with the conduction defects seen in the variant harboring patients [15]. We propose that this variant could explain the cardiac phenotype seen in patient 4, alone or in conjunction with the *TRPM4* variant described below.

The *TRPM4* gene, located on chromosome 19q13.33, encodes for a calcium-activated ion channel highly expressed in the heart, prostate and colon (OMIM 606936) [16]. Variants in the *TRPM4* gene have been associated with ICCD, Brugada syndrome, and heart block. The heterozygous *TRPM4* variant seen in pacemaker patients 4 and 6, p.G844D, has been previously reported in heterozygosity in multiple families with individuals affected by CCD, right bundle branch block (RBBB) or left anterior hemiblock, with variable penetrance. Functional analysis showed that p.G844D significantly increased current amplitudes in transfected HEK293 cells [17, 18].

The *ABCC9* gene maps to chromosome 12p12.1 and encodes the ATP-dependent potassium channel SUR2, which belongs to the family of ABC transporters transporting molecules across membranes (OMIM 601439). Variants in the *ABCC9* gene have been associated with DCM, Cantú syndrome, early repolarization syndrome, Brugada syndrome, myocardial infarction (MI), and atrial fibrillation. The heterozygous *ABCC9* variant (p.V734I) in pacemaker patient 6 has been previously seen in heterozygosity in individuals with early acute MI [19, 20]. In addition, Dr. Charles Antzelevitch’s group has reported the *ABCC9* variant (p.V734I) in four subjects with early repolarization (ERS) and bradycardia. Functional analysis of *ABCC9*-V734I co-expressed with *KCNJ11*-WT in human embryonic kidney cell line TSA201 revealed a gain of function in I_K-ATP_ due to a reduced sensitivity of the ATP-sensitive potassium channel (K_ATP_) to ATP, leading to a five-fold increase in *KCNJ11*-WT function [21]. Moreover, analysis using synchrotron radiation X-ray scattering for the *ABCC9* p.V734I variant also demonstrated that it alters protein-protein interaction, which is critical for the structural integrity of the K_ATP_ channel complex [21]. Interestingly, one index case harboring the *ABCC9* p.V734I variant also harbors a frameshift variant in *SCN5A* (c.3890_3891insA). The patient, a 40 year old male, presented with sinus bradycardia, first degree atrioventricular block (AVB), ventricular bigeminy and a global ER pattern [21]. Taken together, the functional studies, the variant frequency in major databases and the fact that it was identified along with a putative *SCN5A* deleterious variant, we have categorize the *ABCC9* variant p.V734I as 1.2 because it may not be sufficient in isolation to produce a dramatic clinical effect, but in combination with the *TRPM4* variant, it may explain the phenotype of our pacemaker patient 6.

The *APOB* gene maps to chromosome 2p24.1 and encodes the Apo B-100 protein, which regulates the binding and internalization of low density lipoproteins (LDLs) particles (OMIM 107730). Variants in the *APOB* gene have been associated with hypercholesterolemia, hypobetalipoproteinemia, apolipoprotein B deficiency, hypertriglyceridemia, and ischemic stroke. The heterozygous *APOB* variant seen in pacemaker patient 7, p.R3527Q, has been previously seen in heterozygosity in individuals with hypercholesterolemia, MI, and ischemic heart disease (summarized in OMIM 107730). It is known that in rabbit hearts hypercholesterolaemia leads to degeneration of the AV node tissue and the cardiac neural tissue [22]. In addition, aging and hypercholesterolemia in rabbit hearts were associated with the development of atrial tachyarrhythmias [23], while in humans, aging is associated with the increased lipid and collagen deposition around the sinoatrial (SA) node, leading to conduction abnormalities and bradyarrhythmias [24]. However, the role of this variant in the development of conduction disease is currently unknown.

The *SCN5A* gene mapping to chromosome 3p22.2, encodes the alpha subunit Na_v_1.5 of the cardiac voltage-gated sodium channel governing the phase 0 of the cardiac action potential (OMIM 600163). Variants in the *SCN5A* gene have been associated with CCD, SSS, DCM, Brugada syndrome (BrS), long QT syndrome type 3 (LQT3), atrial and ventricular fibrillation, sudden death (adults and infants), arrhythmogenic right ventricular dysplasia, drug-induced arrhythmia, heart block, nodal rhythm defect, and early repolarization syndrome, among others. Previous studies have shown that loss of function variants in *SCN5A*, are usually associated with BrS and ICCD; whereas gain of function variants are usually associated with the LQT3 [25–28]. However, several variants in *SCN5A* have also been associated with more than one clinical phenotype even within the same family [29]. The heterozygous *SCN5A* variant seen in pacemaker patient 7, p.T220I, has been previously seen in an individual with congenital SSS that was compound heterozygote for the *SCN5A* p.T220I and p.R1623X variants (in *trans*). Functional studies on *SCN5A* p.T220I in mammalian cell lines showed a small degree of inactivation delay compared to wild-type cells (the p.R1623X variant encoded non-functional sodium channels, almost inexistent in the plasma membrane) [30, 31]. The p.T220I variant was also found in two relatives with DCM [32], an individual with early onset atrial fibrillation [33], as well as an individual with progressive SND who was a compound heterozygote for the *SCN5A* p.T220I and p.1048SfsX97 variants [34]. Altogether, although the role of the known pathogenic *APOB* variant in the development of SND is currently unknown, it may be possible that the altered lipid metabolism caused by the *APOB* variant could have exacerbated the effect of the *SCN5A* variant. Thus, the combination of the *APOB* variant and the *SCN5A* variant could have been sufficient to exert the effect leading to the cardiac phenotype seen in patient 7. However, further studies are warranted to confirm this hypothesis.

Gourraud and colleagues previously studied familial aggregation of PM implantation in the background of families with inherited form of progressive CCD (PCCD), which suggested a possible genetic involvement in this clinical phenotype [35]. In Gourraud’s study, sequencing of 3 genes (*SCN5A*, *SCN1B*, and *TRPM4*) was performed and one previously reported *SCN5A* variant was detected. In the present report, we have expanded the study of PM implantation irrespective of clinical etiology in the 112 initial patients of our cohort. We found familial aggregation in ICCD and SSS and expanded the genetic analysis to 246 genes using NGS. Overall, of the nine ICCD/SSS pacemaker patients sequenced for 246 genes, three had deleterious variants (category 1.2) in genes (*SCN5A* and *TRPM4*) previously associated with CCD or idiopathic SND [5]. Notably, a category 1.2 variant in *ABCC9* was found in one of the patients harboring the *TRPM4* variant. Besides category 1.1 and 1.2 variants, 44 variants in HGMD were re-categorized as likely genetic modifiers, 59 were re-categorized as VUSs, and 259 were re-categorized to likely not affect function. Although the aforementioned variants, along with the non-HGMD filtered variants (Table C in S1 File), are unlikely to play a significant role in our patients’ phenotype in isolation, they may still cumulatively contribute to the overall clinical presentation or may help reach a threshold for disease manifestation.

## Conclusion

NGS is increasingly being established as a powerful molecular genetics tool for the discovery and detection of variants associated with human disease. We have designed and tested an effective NGS panel that has high sequence depth and low FP and FN rates (average of 4.7% and 1.5%, respectively in splicing and exonic regions with at least 15x sequence depth) for exonic and splicing regions of 246 selected cardiovascular genes. The use of this panel in a small cohort of nine ICCD or SSS individuals with faPPM, along with the detection of a molecular cause in 33% of subjects, suggests that genetic testing in the faPPM population may aid in identifying their genetic etiology and in advising other family members at risk.

## Study Limitations

Our study has several limitations. A major limitation of the study is that we did not have the permission to contact the affected relatives of the family probands to collect clinical information and specimens for the genetic study. In addition to including only retrospective medical record review, the patient population of our pacemaker clinic is primarily of Caucasian ethnicity; thus validating studies will need to be done in other ethnic groups as well. For ethical reasons we could not contact the relative with the implanted pacemaker to verify the implantation, however the index patient did report the age of implantation of his/her relatives during a follow up phone call, increasing the reliability of the information provided. In the validation of the panel reported, we have not performed sequencing runs with the same individual that would allow for analysis of inter-run variability. We are in the process of implementing such analysis for our clinically available panels.

## Supporting Information

S1 Fig ECG of the study patients. We were able to locate ECGs from 5 of the 9 patients studied. Among them, the ECGs of patients 2, 4 and 6 showed complete heart block. ECG of patient 9 showed 2:1 AV block and complete heart block. The ECG of patient 8 showed junctional rhythm with AV dissociation. The ECG of the remaining 4 patients were not available.
(TIF)

S2 Fig Pair-wise comparison of the coverage of 246 target genes. Pearson correlation coefficient of the coverage of 246 target genes was calculated for each pair of the experiment. The red dots represent Pearson correlation coefficients between the expected target region coverage and the observed target region coverage of each experiment/sample; the blue dots show correlation coefficients between pairs of samples. The darker blue color indicates overlapped points, the darker the color, the more number of overlapped points.
(TIF)

S3 Fig Percentage of target bases covered at the indicated coverage thresholds. Each color represents results from the sequence run of a Coriell or pacemaker implanted patient.
(TIF)

S4 Fig Correlation of reads passing filter (PF) with obtained sequence depth. (TIF)

S1 File Includes supplemental patient data; supplemental materials, methods, and patient selection methodology; supplemental results; supplement references, and 12 tables. **Table A.** Exclusion criteria for genetic testing in patients with a first degree relative with a pacemaker. **Table B.** HaloPlex NGS depth of coverage for Coriell and pacemaker samples. **Table C.** Selected HGMD and nonHGMD VUSs in pacemaker patients. **Table D.** Pacemaker variants filtering analysis and classification (number of variants). **Table E.** HGMD variants with disease association in pacemaker patients. **Table F.** Major co-morbidities at the time of pacemaker implantation in patients with ICCD or SSS without structural heart disease. **Table G.** Variant annotation file description. **Table H.** HGMD initial variant classification. **Table I.** Primers for Sanger/Big Dye variant confirmation. **Table J.** HaloPlex intra-run performance. **Table K.** Overall Performance of SNP variant calling in genotype known Coriell samples. **Table L.** Selective analyses of SNP performance in Coriell samples.
(DOCX)

S2 File Includes 3 tables. **Table A.** Pacemaker samples distribution of NGS variants. **Table B.** Pan-cardiovascular panel genes. **Table C.** HaloPlex custom target regions.
(XLSX)
