# Correction: Evaluation of the Genetic Basis of Familial Aggregation of Pacemaker Implantation by a Large Next Generation Sequencing Panel

**DOI:** 10.1371/journal.pone.0147455

**Published:** 2016-01-15

**Authors:** Patrícia B. S. Celestino-Soper, Anisiia Doytchinova, Hillel A. Steiner, Andrea Uradu, Ty C. Lynnes, William J. Groh, John M. Miller, Hai Lin, Hongyu Gao, Zhiping Wang, Yunlong Liu, Peng-Sheng Chen, Matteo Vatta

There are errors in the published S1 File. Please see the correct S1 File here.

## Supporting Information

S1 File Includes supplemental patient data; supplemental materials, methods, and patient selection methodology; supplemental results; supplement references, and 12 tables. Table A. Exclusion criteria for genetic testing in patients with a first degree relative with a pacemaker. Table B. HaloPlex NGS depth of coverage for Coriell and pacemaker samples.Table C. Selected HGMD and nonHGMD VUSs in pacemaker patients. Table D. Pacemaker variants filtering analysis and classification (number of variants). Table E. HGMD variants with disease association in pacemaker patients. Table F. Major co-morbidities at the time of pacemaker implantation in patients with ICCD or SSS without structural heart disease. Table G. Variant annotation file description. Table H. HGMD initial variant classification. Table I.Primers for Sanger/Big Dye variant confirmation. Table J. HaloPlex intra-run performance.Table K. Overall Performance of SNP variant calling in genotype known Coriell samples. Table L. Selective analyses of SNP performance in Coriell samples.
(DOCX)
