# Supplementary figures and images for: Evaluation of the Genetic Basis of Familial Aggregation of Pacemaker Implantation by a Large Next Generation Sequencing Panel
Source: PLoS One. 2015 Dec 4;10(12):e0143588. doi: 10.1371/journal.pone.0143588 (PMC4670209; doi:10.1371/journal.pone.0143588)

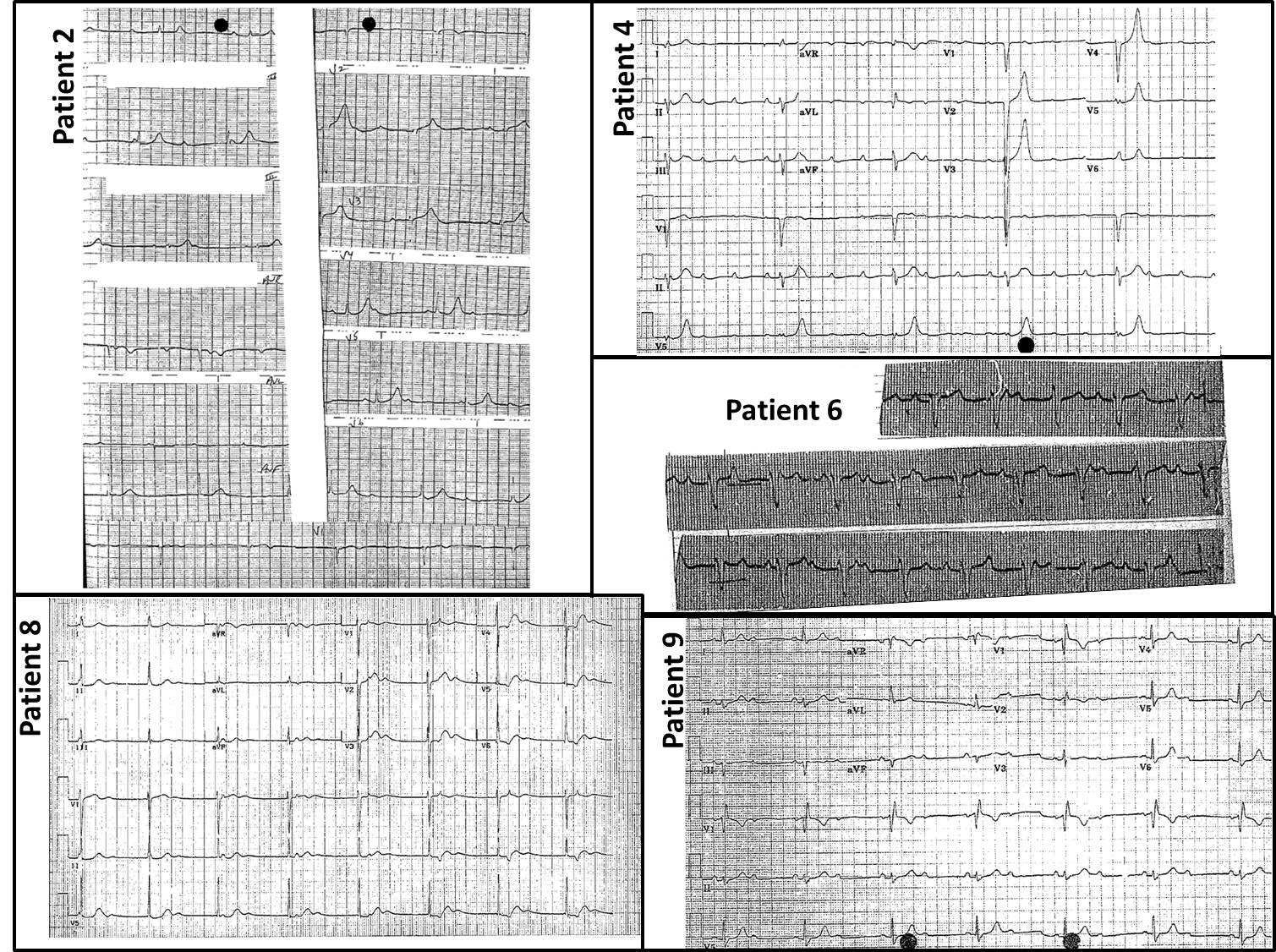

Supplement: S1 Fig — We were able to locate ECGs from 5 of the 9 patients studied. Among them, the ECGs of patients 2, 4 and 6 showed complete heart block. ECG of patient 9 showed 2:1 AV block and complete heart block. The ECG of patient 8 showed junctional rhythm with AV dissociation. The ECG of the remaining 4 patients were not available. (TIF) [file pone.0143588.s001.tif]

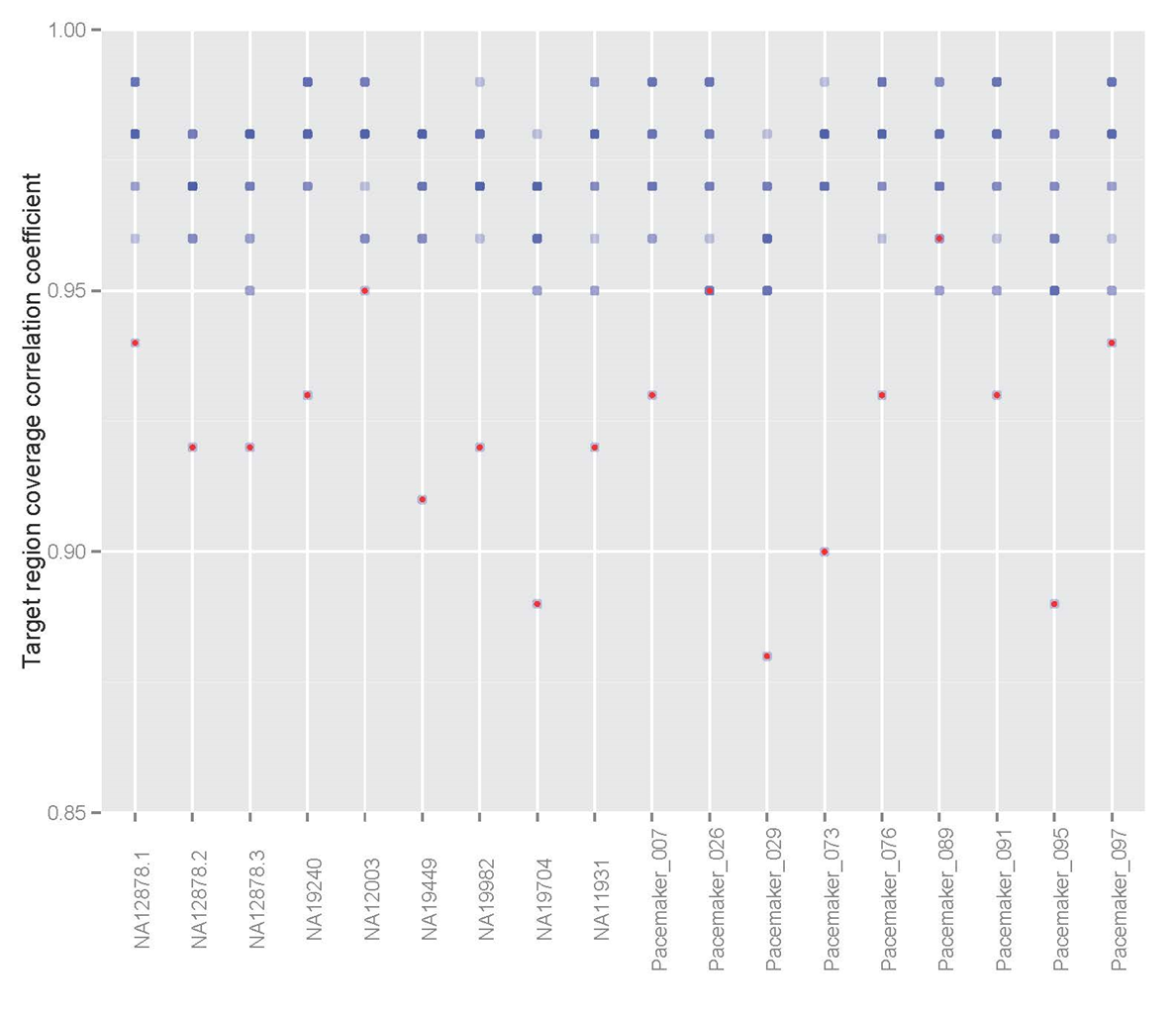

Supplement: S2 Fig — Pearson correlation coefficient of the coverage of 246 target genes was calculated for each pair of the experiment. The red dots represent Pearson correlation coefficients between the expected target region coverage and the observed target region coverage of each experiment/sample; the blue dots show correlation coefficients between pairs of samples. The darker blue color indicates overlapped points, the darker the color, the more number of overlapped points. (TIF) [file pone.0143588.s002.tif]

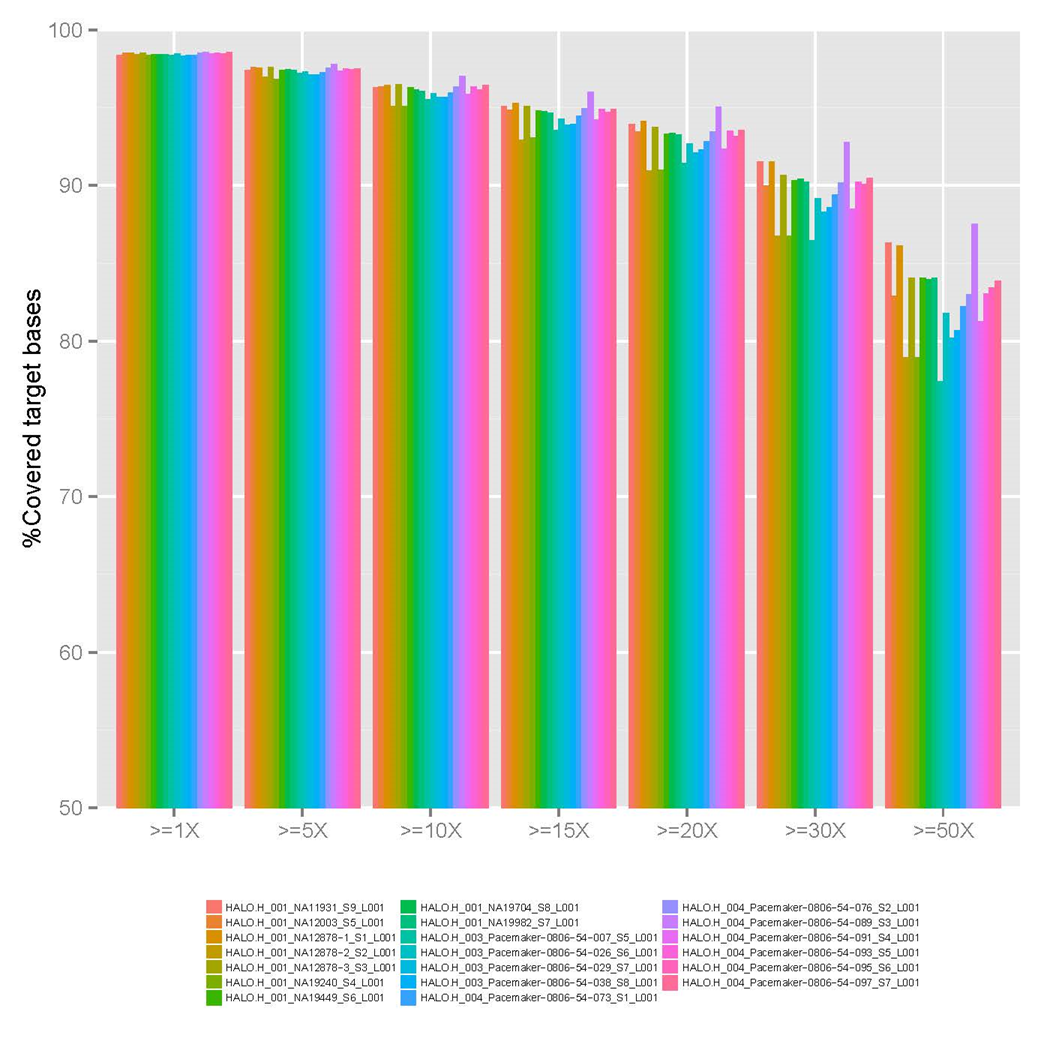

Supplement: S3 Fig — Each color represents results from the sequence run of a Coriell or pacemaker implanted patient. (TIF) [file pone.0143588.s003.tif]

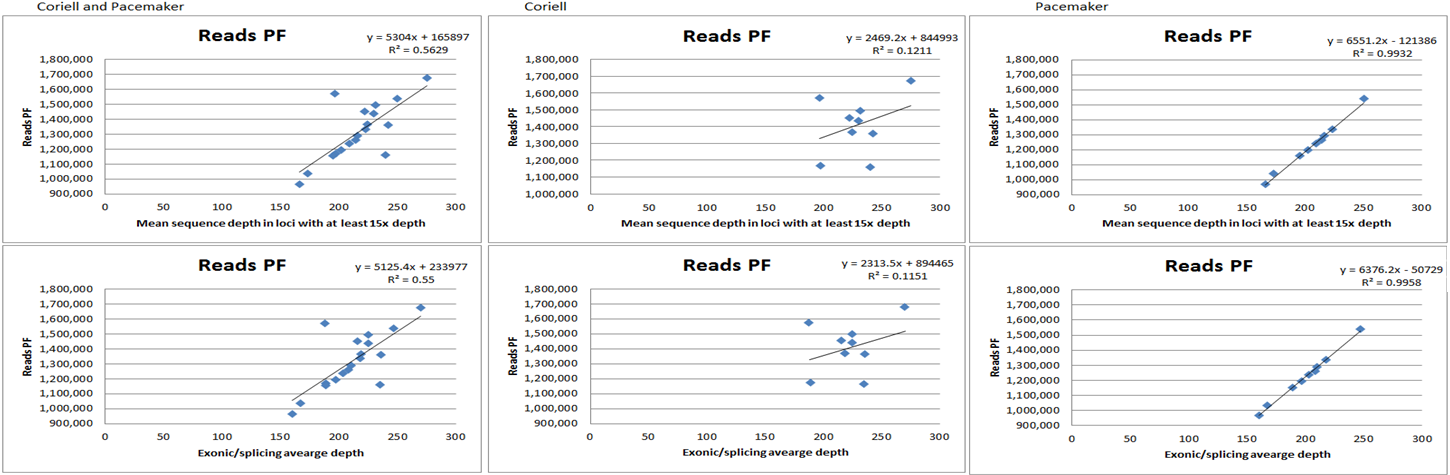

Supplement: S4 Fig — (TIF) [file pone.0143588.s004.tif]
