# Supplementary material for: Evaluation of the Genetic Basis of Familial Aggregation of Pacemaker Implantation by a Large Next Generation Sequencing Panel
Source: PLoS One. 2015 Dec 4;10(12):e0143588. doi: 10.1371/journal.pone.0143588 (PMC4670209; doi:10.1371/journal.pone.0143588)
Supplement: S1 File — Table A. Exclusion criteria for genetic testing in patients with a first degree relative with a pacemaker. Table B. HaloPlex NGS depth of coverage for Coriell and pacemaker samples. Table C. Selected HGMD and nonHGMD VUSs in pacemaker patients. Table D. Pacemaker variants filtering analysis and classification (number of variants). Table E. HGMD variants with disease association in pacemaker patients. Table F. Major co-morbidities at the time of pacemaker implantation in patients with ICCD or SSS without structural heart disease. Table G. Variant annotation file description. Table H. HGMD initial variant classification. Table I. Primers for Sanger/Big Dye variant confirmation. Table J. HaloPlex intra-run performance. Table K. Overall Performance of SNP variant calling in genotype known Coriell samples. Table L. Selective analyses of SNP performance in Coriell samples. (DOCX) [file pone.0143588.s005.docx]

Evaluation of the Genetic Basis of Familial Aggregation of Pacemaker Implantation by a Large Next Generation Sequencing Panel

**Short title:** Cardiovascular NGS for faPPM Evaluation

Patrícia B. S. Celestino-Soper^1¶^, Anisiia Doytchinova^2¶^, Hillel A. Steiner^2,4,5^, Andrea Uradu^2^, Ty C. Lynnes^1^, William J. Groh^2^, John M. Miller^2^, Hai Lin^1,3^, Hongyu Gao^1^, Zhiping Wang^3^, Yunlong Liu^1,3^, Peng-Sheng Chen^2^, Matteo Vatta^1,2*^

**1** Department of Medical and Molecular Genetics, Indiana University School of Medicine, Indianapolis, IN, USA, **2** Krannert Institute of Cardiology, Division of Cardiology, Department of Medicine, Indiana University School of Medicine, Indianapolis, IN, USA, **3** Center for Computational Biology and Bioinformatics, Indiana University Purdue University Indianapolis, Indianapolis, IN, USA, **4** Baruch Padeh Medical Center, Poriya MP Lower Galilee, Israel, **5** Faculty of Medicine in the Galilee, Bar-Ilan University, Safed, Israel

*** Corresponding author:**

Matteo Vatta, PhD, FACMG

550 University Blvd, UH AOC 6029, Indianapolis, IN 46202

Phone 317 944-1066, Email: mvatta@iu.edu

^¶^  These authors contributed equally to this work.

**Supplemental patient data: patients selected for disease-targeted NGS panel**

All patients with implanted pacemakers prospectively presenting to routine follow up at the pacemaker clinics of the Krannert Institute of Cardiology of Indiana University, Indianapolis, IN, between September of 2008 and April of 2009 were requested to participate. Patients with implantable cardioverter-defibrillators were excluded. After giving written informed consent, the subjects were asked to fill a detailed questionnaire regarding ethnicity as well as place of birth of grandparents, number of siblings and children, and details of other first degree family relatives (FDR) with implanted permanent pacemakers. Clinical data regarding indication for pacemaker implantation, co-morbidities, other arrhythmias, medications, and age at implant were retrospectively collected from the medical records. Pacemaker dependency was defined as lacking any ventricular escape rhythm when the device was programmed to a lower rate of ventricular pacing at 30 beats per minute. Telephone interviews were conducted to further confirm the number of FDRs with implanted pacemakers.

A second chart review was performed in all patients who reported having FDR with a pacemaker. Only patients with a FDR with pacemaker and suspected congenital heart block in the absence of congenital heart disease or patients with FDR with a pacemaker and ICCD or SSS without structural heart disease as determined by clinical notes and/or cardiovascular imaging before or within a month after PPM implantation were selected for genetic analysis. ICCD was defined as absence of structural heart disease (specifically the presence of obstructive coronary artery disease or history of myocardial infarction, any congenital heart disease - excluding congenital heart block -, cardiomyopathy, left ventricular hypertrophy and ≥ moderate valvular disease or history of valvular replacement or repair) or a secondary cause resulting or implicated in the rhythm disturbance. Patients with left atrial enlargement, valvular thickening without stenosis, ≤ moderate mitral annular calcification and/or mild valvular regurgitation in the absence of other abnormalities were selected for genetic analysis because in the opinion of the treating clinicians these findings alone were insufficient to explain the need for device implantation. One patient had mild aortic dilatation noted on one echocardiogram, but not reported on subsequent echocardiograms and was also selected for genetic analysis. Retrospective follow up data in patients with ICCD or SSS without structural heart disease after pacemaker insertion was also collected from the medical chart.

In total, 112 patients (59 men) were enrolled, 86.6% of whom were of Caucasian ethnicity. Among them, 24 had at least one FDR with an implanted pacemaker (21.4%) and 4 (3.6%) had multiple FDRs (range 2-4) with implanted pacemakers. In all but one of the 24 patients, there were sufficient clinical records to determine if the subject was a suitable candidate for genetic analysis. A total of 19 (83%) out of the 23 patients had at least one form of cardiovascular imaging available before PPM insertion. In most cases (n=18) this was an echocardiogram completed within -2.2±5.1 (range 0 to -21) months of PPM insertion. A total of 8/23 (34.8%) subjects had a cardiac catheterization within -4.4±10.8 (range 0 to -31) months of PPM insertion and 5/23 (21.7%) had an imaging stress test available within -0.2±0.4 (range 0 to -1) months of PPM insertion. Of the remaining four subjects who had no cardiovascular imaging available prior to PPM insertion, two had suspected congenital heart block in the absence of congenital heart disease and two had clinical notes clearly describing the presence of structural heart disease or a secondary cause (postoperative complete heart block) prior to PPM insertion. Nine out of the 23 patients (39.1%) with ICCD or SSS without structural heart disease were determined to be suitable for genetic analysis. This constituted 8% of the study population (summarized in Table 1). DNA aliquots from each of the 9 individuals (pacemaker patients 1-9) with implanted pacemaker and family history of pacemaker implantation selected for genetic testing were obtained from the Indiana Biobank using the Gentra Puregene Blood kit (Qiagen, Germantown, MD) and were tested by NGS using our custom HaloPlex pan-cardiovascular panel for investigation of a possible genetic component to their clinical presentation. Below is a description of each patient at the time of their study enrollment and a summary of their clinical course as available in the medical records.

**Patient 1:** The patient is a 65 year old white male with a history of chronic obstructive pulmonary disease, hypertension, alcohol and tobacco abuse, seizures, chronic hyponatremia, and lactunar infarcts who had received a PPM at age 64. At that time, he was found down by a bystander and admitted to the hospital for further care. Electrocardiogram (ECG) revealed intermittent complete heart block with junctional escape. He was hypotensive and required transvenous pacemaker. Subsequent ECGs showed sinus rhythm with marked PR delay (PR>400 ms), and periods of high degree AV block. Thyroid stimulating hormone levels and electrolytes were normal. Echocardiogram revealed only mildly thickened aortic valve without stenosis and moderate mitral annular calcification, but was otherwise normal. No cause for his conduction disturbance was identified and the patient received a PPM. He reported that his father also required a PPM. A follow up of 4.4 years after PPM implant was available for this patient. He was not PPM dependent and follow-up electrophysiology notes revealed that his complete heart block had resolved. Subsequent echocardiograms demonstrated stage 1 diastolic dysfunction and pulmonary hypertension, although he did not have clinical symptoms of heart failure. There are conflicting records as to whether or not he developed coronary artery disease but there is no definite evidence of coronary artery disease in the medical records. In follow up however, he developed strokes, carotid artery stenosis which required carotid endarterectomy, and recurrent chronic obstructive pulmonary disease exacerbations with respiratory failure, aspiration pneumonias and malnutrition. Near the end of his life, the patient was transferred to the palliative care service.

**Patient 2:** The patient is a 48 year old white female who had complete heart block for many years prior to her presentation. The etiology of her conduction disturbance was unknown; however it was attributed to either congenital complete heart block or a sequela of scarlet fever at the age of 1. She had a PPM implanted at age 24 for symptomatic bradycardia in the setting of third degree heart block. She reported that her sister also required PPM insertion. Our records begin when she presented for electrophysiology follow up at age 33, at which time she had no other past medical history and took no medications. Her underlying rhythm on repeated PPM interrogations was sinus with complete heart block and at times no ventricular escape was noted. Per subsequent medical records it was noted that the patient’s mother had Huntington’s chorea and the patient’s child had a history of mental retardation. A total of 29 years of follow up was available for this patient after PPM insertion. During this follow up she was noted to exhibit some signs of low IQ and mental retardation; however, her competency for medical decision making was not in question. She did not display any signs of Huntington’s chorea. She developed angiolipomas, which were believed to be familial, hypertension, diabetes mellitus type 2, obstructive sleep apnea, chronic obstructive pulmonary disease as well as coronary artery disease requiring percutaneous intervention at age 44. She then required repeated coronary interventions and developed congestive heart failure with mild left ventricular systolic dysfunction.

**Patient 3:** The patient is a 71 year old African-American male who underwent a PPM insertion at age 64 for symptomatic bradycardia and chronotropic incompetence. At that time his only medical problems were hyperlipidemia, anxiety and depression. He had presented to the emergency department several times for chest pain and syncope. Electrocardiograms showed sinus bradycardia. Dobutamine stress and resting echocardiogram were negative for ischemia and structural heart disease other than left atrial dilatation with a diameter of 4.18 cm (normal 2.1-3.7 cm), but significant for blunted heart rate response to stress. Cardiac catheterization revealed normal coronary arteries and normal ejection fraction. A treadmill electrocardiogram confirmed the diagnosis of chronotropic incompetence. The patient reported a family history of a sister who required PPM insertion. A total of 12 years of follow up after PPM insertion was available for this patient. He was not pacemaker dependent. During this follow up, he developed biopsy proven lupus nephritis, hypertension, chronic obstructive pulmonary disease and ischemic cerebrovascular accident. He had no evidence of coronary artery disease or clinical congestive heart failure although he did develop left ventricular hypertrophy and stage 1 diastolic dysfunction on subsequent echocardiograms.

**Patient 4:** The patient is a 66 year old white male who underwent PPM insertion 10 years prior to his study enrollment for complete heart block. At that time he had no known past medical history other than ongoing tobacco abuse and history of heavy ethanol use in the past. He presented with acute symptoms of dyspnea, nausea and diaphoresis at rest. Electrocardiogram revealed left bundle branch block and complete heart block with an escape rhythm at 30 beats per minute. He required temporary pacing and ultimately a PPM insertion. During this admission, his cardiac catheterization revealed normal coronary arteries and echocardiogram did not demonstrate any abnormalities. No other cause for the conduction disturbance was identified. He reported that his father also required PPM insertion. A total of 15 years of follow up after PPM insertion was available for this patient. He was noted to be pacemaker dependent. Repeated stress echocardiogram 5 years after PPM insertion obtained for syncope and dyspnea demonstrated a left anterior descending coronary artery wall motion abnormality which was unchanged with stress and an abnormal apical motion on post stress images possibly due to pacing. A subsequent echocardiogram however did not demonstrate any wall motion abnormalities and was only significant for stage 1 diastolic dysfunction and dyssynchronous contraction of the apical inferior segment. He remains only on aspirin and pravastatin as his sole medications and continues to do clinically well with no evidence of coronary artery disease or congestive heart failure symptoms.

**Patient 5:** The patient is a 77 year old white male with history of PPM insertion at age 71. At that time his medical co-morbidities included hypertension and hypothyroidism. He presented to the emergency department with pre-syncope and electrocardiogram revealed Mobitz II second degree atrioventricular block. Echocardiogram was only significant for mild left atrial enlargement of 3.7 cm (normal 2.1-3.7 cm) and minimal mitral and tricuspid valve regurgitation. He reported that his father had undergone PPM implantation. A total of 8.9 years of follow up after PPM insertion was available for this patient. He was not noted to be pacemaker dependent. Three years after his PPM insertion the patient suffered from non-ST elevation myocardial infarction due to single vessel coronary artery disease and required percutaneous coronary intervention. Echocardiogram revealed normal systolic function and stage 1 diastolic dysfunction. He subsequently developed ischemic stroke with hemorrhagic conversion, carotid artery disease and seizures. He suffered a pulseless electrical activity cardiopulmonary arrest at age 80 from which he could not be resuscitated successfully and expired.

**Patient 6:** The patient is an 81 year old white male who received a PPM for symptomatic 2:1 atrioventricular block at age 68. At that time he did not have any medical co-morbidities and his only medication was aspirin. His echocardiogram was normal other than findings of mild aortic and mitral regurgitation and thickened mitral valve without stenosis. He reported that his father also required PPM insertion. A total of 17.3 years of follow up after PPM insertion was available for this patient. He was pacemaker dependent and his conduction disturbance progressed to complete heart block. During this time, exercise myocardial perfusion imaging showed a fixed inferior wall defect, however this was not detected on subsequent stress echocardiogram and likely represented an artifact. He did not develop any signs of congestive heart failure or coronary artery disease and only suffered from elevated blood pressure. He remained only on aspirin and losartan as his sole medications.

**Patient 7:** The patient is a 59 year old white female with history of congenital complete heart block who received PPM at age 24 for symptomatic bradycardia. Her first records in our system began at age 49. At that time, there is no mention of congenital heart disease or other medical co-morbidities. She was also not taking any medications. While a complete echocardiogram report was not available in our records at that time, electrophysiology notes list that her left ventricular systolic function was normal. The reason for the congenital heart block was not known. She reported that her father also required PPM insertion. A total of 35.8 years of follow up after PPM insertion was available for this patient. She was not pacemaker dependent. After the age of 53 she developed hypertension, diabetes, hyperlipidemia, and coronary artery disease requiring coronary artery bypass grafting. She was also noted to have atrial flutter and atrial fibrillation at age 60.

**Patient 8:** The patient is a 69 year old white female who received a PPM at age 53 for sick sinus syndrome. At that time, her only medical problems were hypertension which was treated with verapamil for 3-4 years, hyperlipidemia, not on therapy, and supraventricular tachycardia, believed to be due to atrial tachycardia. During the supraventricular episodes, the patient’s heart rate was as high as 180 beats per minute, which was associated with fatigue, generalized weakness, and gradual decrease in her exercise tolerance. The episodes would usually terminate with a pause. She was also documented to have frequent runs of premature ventricular contractions. Attempts to treat the supraventricular tachycardia with beta blockers were not well tolerated due to medication side effects and treatment with digoxin resulted in profound bradycardia with a junctional escape rhythm. For these reasons she underwent PPM insertion. Echocardiogram report from an outside institution prior to PPM insertion per our records revealed dilated left atrium of 5.0 cm and mild aortic and tricuspid insufficiency with an estimated right ventricular systolic pressure of 40 mmHg. The left ventricle measured 5.5 cm per report, however, no details were provided in the report of where these measurements were taken and original images were not available. She reported that her son also required PPM and suffered from atrial fibrillation. Medical records revealed that her father died at age 29 from an unknown congenital heart defect. A total of 20.7 years of follow up after PPM insertion was available for this patient. She was not PPM dependent. She developed atrial fibrillation at age 71 and also suffered from constipation, hematuria, intermittent vaginal bleeding, benign adrenal myelolipoma, and diffuse arthritis requiring multiple knee and back surgeries. She did not develop any symptom of congestive heart failure or coronary artery disease. Records reveal that she had a negative stress test at age 66, which was obtained for atypical chest pain. This study specifically revealed normal left ventricular size and function.

**Patient 9:** The patient is a 73 year old African-American female who received a PPM at age 59 for symptomatic bradycardia due to Mobitz II heart block. At that time she suffered from hypertension and active alcohol abuse with history of alcohol withdraw. She presented to her primary care physician with complaints of dizziness on standing, decreased exercise tolerance and paroxysmal nocturnal dyspnea. Her electrocardiogram revealed sinus bradycardia and Mobitz type I heart block for which she hospitalized due to her symptomatology. During that hospital stay she had an echocardiogram showing normal left ventricular systolic function, no wall motion abnormalities, mildly dilated aorta (not detected on subsequent echocardiograms), and mild mitral and tricuspid valve regurgitation. She also underwent a tilt test which was positive for syncope and the development of Mobitz type II heart block. No reversible causes were identified for patient’s conduction disturbances and she received a PPM. She reported a family history of her sister and brother both requiring PPM. A total of 20 years of follow up was available for this patient after PPM insertion. She was not PPM dependent. During this time she developed atrial tachycardia which required ablation and later, suffered from dementia and osteoporosis. There are no records of the patient developing congestive heart failure symptoms or coronary artery disease.

**Supplemental materials, methods, and patient selection methodology**

**Panel design**

Table B in S2 File contains a list of all 246 cardiovascular related genes whose coding regions were targeted for enrichment using the Agilent HaloPlex Target Enrichment System (Agilent Technologies, Santa Clara, CA). Exon coordinates for all 246 genes were obtained from RefSeq UCSC hg19. Following, a minimum of 50 bp 5’ and 15 bp 3’ from each exon was added in order to include branch-site sequences, which could harbor pathogenic variants [1]. Targeted regions that were found to have corresponding pseudogenes elsewhere in the genome were excluded. The resulting target coordinates were uploaded into the Agilent SureDesign web tool for probe design (Table C in S2 File) [2]. The final design consisted of a total of 4227 regions comprising 1.035 Mbp with an expected coverage of 99.1% of target regions (see Table B in S2 File for expected coverage per gene). The total sequenceable design size obtained was of 2.37 Mbp and the recommended minimum sequencing per sample was of 474 Mbp for 200x sequencing coverage depth.

**Library preparation with target enrichment**

Library preparation followed manufacturer’s instructions using the Agilent HaloPlex Target Enrichment System for Illumina Sequencing protocol version D.5 (Agilent Technologies, Santa Clara, CA). Briefly, genomic DNA was quantified using Qubit 2.0 Fluorometer (Life Technologies, Carlsbad, CA) and diluted to obtain 225 ng of each sample at a final concentration of 5 ng/ul. Following DNA digestion, validation of restriction enzyme activity was performed using an Agilent 2200 TapeStation (Agilent Technologies, Santa Clara, CA). The digested DNA was hybridized to HaloPlex custom probes for target enrichment and sample indexing via incubation for 16 hours at 54°C using a Veriti Thermal Cycler (Life Technologies, Carlsbad, CA). Target DNA-HaloPlex probe hybrids containing biotin were captured using streptavidin magnetic beads, and target DNA was subsequently eluted from probes with NaOH. Captured target libraries were amplified by PCR using a Herculase II Polymerase (Agilent Technologies, Santa Clara, CA) in a Veriti Thermal Cycler. Finally, PCR amplified libraries were purified using an Agencourt AMPure XP beads (Beckman Coulter, Brea, CA) followed by elution with Tris-HCl buffer (pH 8.0).

**Sequencing**

Enrichment was validated and target DNA concentration of 175-625 bp size products was determined using the Agilent 2200 TapeStation. Eleven samples were pooled per run in equimolar amounts of 4.5 nM each and the pooled library was reassessed with the 2200 TapeStation. Approximately 3 pM of pooled libraries spiked with 5% of 12.5 pM PhiX library (Illumina, Inc., San Diego, CA) were loaded onto an Illumina MiSeq using a standard reagent kit v2 (300 cycles) and standard flow cell. Sequencing was performed using 151 + 151 bp paired-end protocol and single indexing with 8-nt index read.

**Bioinformatics pipeline and data analysis**

FASTQ files were obtained from the MiSeq instrument for each run. Adapter sequences and low quality bases (trim from the 3’ end) were trimmed from the 150 bp pair-end MiSeq reads using Trim Galore (v0.3.2). The resulting reads were mapped to the human reference genome UCSC build hg19 using BWA version 0.7.5a and SAMtools version 0.1.19 [3, 4]. The aligned reads were further realigned and the quality scores were recalibrated using GATK 2.8-1 [5]. Variants were called using GATK Unified Genotyper restricted to specified target regions. Variants were annotated using ANNOVAR and the Human Gene Mutation Database (HGMD) [6-10].

Additional data analysis was performed with in-house written Perl scripts or conducted in R [11]. Figures were generated using ggplot2 in R [12]. A final report of variants was generated per sample for the targeted gene regions. The report consists of variant mapping information, gene annotation, amino acid change annotation, variant functional annotation (SIFT, PolyPhen, LRTs, and Mutation Taster), variant evolutionary conservation annotation (PhyloP and GERP++), variant presence and allele frequencies in currently publicly sequenced populations (dbSNP identifiers, 1000 Genomes Project allele, NHLBI-ESP 6500 exome project), and known disease-related functional annotation from HGMD (see Table G).

**Table G: Variant annotation file description.**

| **Column names** | **Explanation** | **Example** | **Note** |
| --- | --- | --- | --- |
| HGMD_search | Variant genomic coordiantes in HGMD (Human Gene Mutatio database), the format is chromosome:position | chr1:977330 |  |
| HGMD_mutation | HGMD mutation | ins 1 bp codon 53, Ala45Thr |  |
| HGMD_Variant_Class | HGMD variant class, functional annotation of a variant | DM, DP, FP, DFP, FTV | **DM** = Disease causing mutation, pathological mutations reported to be disease causing in the original literature report.  **DM?** = Likely disease causing (likely pathological) mutation. **DP** = Disease associated polymorphism. These are reported to be in significant association with disease (p<0.05) and are assumed to be functional (e.g. as a consequence of location, evolutionary conservation, replication studies etc), although there may be as yet no direct evidence (e.g. from an expression study) of function.  **FP** = Polymorphism affecting the structure, function or expression of a gene but with no disease association reported yet.  **DFP** = Disease associated polymorphism with additional supporting functional evidence. These are reported to be in significant association with disease (p<0.05) and to have evidence for being of direct functional importance (e.g. as a consequence of altered expression, mRNA studies etc). **FTV** = Frameshift or truncating variant with no disease association reported yet. |
| CHROM | Chromosome, an identifier from the reference genome | chr1 | GATK vcf file |
| POS | The reference position, with the 1st base having position 1. Positions are sorted numerically, in increasing order, within each reference sequence CHROM.   It is permitted to have multiple records with the same POS. | 977330 | GATK vcf file |
| rsID | Variant dbSNP ID | rs2799066 | GATK vcf file |
| REF | Reference base(s) at the position Each base must be one of A,C,G,T,N (case insensitive). Multiple bases are permitted. The value in the POS field refers to the position of the first base in the String. For simple insertions and deletions in which either the REF or one of the ALT alleles would otherwise be null/empty, the REF and ALT Strings must include the base before the event (which must be reflected in the POS field), unless the event occurs at position 1 on the contig in which case it must include the base after the event; this padding base is not required (although it is permitted) for e.g. complex substitutions or other events where all alleles have at least one base represented in their Strings. If any of the ALT alleles is a symbolic allele (an angle-bracketed ID String "<ID>") then the padding base is required and POS denotes the coordinate of the base preceding the polymorphism. | T | GATK vcf file |
| ALT | Alternate non-reference alleles at the position, Options are base Strings made up of the bases A,C,G,T,N, (case insensitive) or an angle-bracketed ID String (”<ID>”) or a breakend replacement string as described in the section on breakends. If there are no alternative alleles, then the missing value should be used. | C | GATK vcf file |
| QUAL | Variant Quality (QUAL = -10 * log(probability that ALT is incorrect), the higher the QUAL score, the more confident we are about the variants | 1900.77 | GATK vcf file; QUAL is indeed phred-scaled |
| FILTER* | PASS if this position has passed all filters. If filters have not been applied, then this field should be set to the missing value. | . | GATK vcf file |
| AC | allele count in genotypes, for each ALT allele, in the same order as listed | 2 | GATK vcf file |
| AF | allele frequency for each ALT allele in the same order as listed: use this when estimated from primary data, not called genotypes | 1 | GATK vcf file |
| AN | total number of alleles in called genotypes | 2 | GATK vcf file |
| BaseQRankSum | Z-score from Wilcoxon rank sum test of Alt Vs. Ref base qualities | NA | GATK vcf file |
| DB | dbSNP membership | NA | GATK vcf file |
| DP | Approximate read depth at POS; some low qualtiy reads may have been filtered | 73 | GATK vcf file |
| DS* | Were any of the samples downsampled? Not applicable here. | NA | GATK vcf file |
| Dels | Fraction of Reads Containing Spanning Deletions | 0 | GATK vcf file |
| FS | Phred-scaled p-value using Fisher's exact test to detect strand bias | 0 | GATK vcf file |
| HRun | Largest Contiguous Homopolymer Run of Variant Allele In Either Direction | 2 | GATK vcf file |
| HaplotypeScore | Consistency of the site with at most two segregating haplotypes | 0 | GATK vcf file |
| InbreedingCoeff* | Inbreeding coefficient as estimated from the genotype likelihoods per-sample when compared against the Hardy-Weinberg expectation | NA | GATK vcf file |
| MLEAC | Maximum likelihood expectation (MLE) for the allele counts (not necessarily the same as the AC), for each ALT allele, in the same order as listed | 2 | GATK vcf file |
| MLEAF | Maximum likelihood expectation (MLE) for the allele frequency (not necessarily the same as the AF), for each ALT allele, in the same order as listed | 1 | GATK vcf file |
| MQ | RMS Mapping Quality | 60 | GATK vcf file |
| MQ0 | Total Mapping Quality Zero Reads | 0 | GATK vcf file |
| MQRankSum | Z-score From Wilcoxon rank sum test of Alt vs. Ref read mapping qualities | NA | GATK vcf file |
| QD | Variant Confidence/Quality by Depth | 26.04 | GATK vcf file |
| ReadPosRankSum | Z-score from Wilcoxon rank sum test of Alt vs. Ref read position bias | NA | GATK vcf file |
| GT | Genotype |  | GATK vcf file |
| AD | Allelic depths for the ref and alt alleles in the order listed | 0,73 | GATK vcf file |
| GQ | Genotype Quality | 99 | GATK vcf file |
| PL | Normalized, Phred-scaled likelihoods for genotypes | 1929,138,0 | GATK vcf file |
| Target | Used to indicate if a variant is on the panel target regions or not. 1 indicates on target and 0 off target | 1 |  |
| Damage | Number of total deleterious records (max 6) on LJB2_SIFT (<0.05), LJB2_PolyPhen2_Pred (D/P), LJB2_LRT_Pred (D), LJB2_MutationTaster_Pred (A/D), LJB_MutationAssessor_Pred (high/medium), LJB2_FATHMM (<-1.5) | 0 |  |
| Func_refGene | Where the mutation is located based on the reference gene annotation, eg. Intronic, exonic, UTR5, UTR3, splicing, intergenic, upstream, etc. | intronic | ANNOVAR annotation |
| Gene_refGene | Reference gene name | AGRN | ANNOVAR annotation |
| ExonicFunc_refGene | Functional annotation of mutation, eg. NA, Synonymous, nonsynonymous, stopgain, stoploss, frameshift insertion, etc. | NA | ANNOVAR annotation |
| AAChange_refGene | Amino acid change resulted in. | NA | ANNOVAR annotation |
| phastConsElements46way | Multiple alignments of 45 vertebrate genomes to the human genome. ANNOVAR uses phastCons 46-way alignments to annotate variants that fall within conserved genomic regions. It contains two pieces of information: Score and Name. Score is the normalized score assigned by UCSC Genome Browser, and this score range from 0 to 1000 for the sole purpose of having a standard range of values to display in browser. The "Name=lod=x" is used to tell the user a name for the region. | Score=553;Name=lod=235 | ANNOVAR annotation |
| genomicSuperDups | To identify variants located in segmental duplications. Genetic variants that are mapped to segmental duplications are most likely sequence alignment errors and should be treated with extreme caution. The "Name" field in the output represents the other "matching" segment in genome (which is located in the same chromosome at chr1:1567836). The "Score" field is the sequence identity with indels between two genomic segments. | Score=0.990441;Name=chr1:1567836 | ANNOVAR annotation |
| esp6500si_all | alternative allele frequency in all subjects in the NHLBI-ESP project with 6500 exomes | 0.852593 | ANNOVAR annotation |
| esp6500si_ea | alternative allele frequency in European Americans in the NHLBI-ESP project with 6500 exomes | 0.920777 | ANNOVAR annotation |
| esp6500si_aa | alternative allele frequency in African Americans in the NHLBI-ESP project with 6500 exomes | 0.719446 | ANNOVAR annotation |
| 1000g2012apr_all | alternative allele frequency data in 1000 Genomes Project | 0.89 | ANNOVAR annotation |
| 1000g2012apr_eur | alternative allele frequency data in 1000 Genomes Project (european) | 0.93 | ANNOVAR annotation |
| 1000g2012apr_amr | alternative allele frequency data in 1000 Genomes Project (admixed american) | 0.93 | ANNOVAR annotation |
| LJB2_SIFT | whole-exome LJBSIFT scores(version 2). In the updated version 2 (ljb2_sift), the scores were now the SIFT score itself. This means a variant with score<0.05 is predicted as deleterious. | NA | ANNOVAR annotation |
| LJB2_PolyPhen2_HDIV | whole-exome PolyPhen scores built on HumanDiv database (for complex phenotypes). ljb2_pp2hdiv should be used when evaluating rare alleles at loci potentially involved in complex phenotypes, dense mapping of regions identified by genome-wide association studies, and analysis of natural selection from sequence data. The authors recommend calling "probably damaging" if the score is between 0.957 and 1, and "possibly damaging" if the score is between 0.453 and 0.956, and "benign" is the score is between 0 and 0.452. | NA | ANNOVAR annotation |
| LJB2_PP2_HDIV_Pred | "D" ("probably damaging"), "P" ("possibly damaging") and "B" ("benign"). | NA | ANNOVAR annotation |
| LJB2_PolyPhen2_HVAR | whole-exome PolyPhen version 2 scores built on HumanVar database (for Mendelian phenotypes). ljb2_pp2hvar should be used for diagnostics of Mendelian diseases, which requires distinguishing mutations with drastic effects from all the remaining human variation, including abundant mildly deleterious alleles.The authors recommend calling "probably damaging" if the score is between 0.909 and 1, and "possibly damaging" if the score is between 0.447 and 0.908, and "benign" is the score is between 0 and 0.446. | NA | ANNOVAR annotation |
| LJB2_PolyPhen2_HVAR_Pred | "D" ("probably damaging"), "P" ("possibly damaging") and "B" ("benign"). | NA | ANNOVAR annotation |
| LJB2_PolyPhen2_Pred | combination of LJB2_PP2_HDIV_Pred and LJB2_PolyPhen2_HVAR_Pred, take the more deleterious one | NA | ANNOVAR annotation |
| LJB2_LRT | whole-exome LRT scores. The score ranges from 0 to 1 and a larger score signifies that the codon is more constrained or a NS is more likely to be deleterious. | NA | ANNOVAR annotation |
| LJB2_LRT_Pred | D: deleterious; N, neutral | NA | ANNOVAR annotation |
| LJB2_MutationTaster | whole-exome MutationTaster scores. The score ranges from 0 to 1 and a larger score means more likely to be deleterious. | NA | ANNOVAR annotation |
| LJB2_MutationTaster_Pred | There are four possible predictions: "A" ("disease_causing_automatic"), "D" ("disease_causing"), "N" ("polymorphism") or "P" ("polymorphism_automatic"). | NA | ANNOVAR annotation |
| LJB_MutationAssessor | whole-exome MutationAssessor scores (version 2). It ranges from -5.545 to 5.975; the larger the score the more likely it will be deleterious. | NA | ANNOVAR annotation |
| LJB_MutationAssessor_Pred | There are two possible predictions: predicted functional (high, medium), predicted non-functional (low, neutral)" | NA | ANNOVAR annotation |
| LJB2_FATHMM | whole-exome FATHMM scores [21]. It ranges from -18.09 to 11.0; the smaller the score the more likely it will be deleterious. If a score is smaller than -1.5 the corresponding NS is predicted as "D(AMAGING)"; otherwise it is predicted as "T(OLERATED)". | NA | ANNOVAR annotation |
| LJB2_GERP | whole-exome GERP++ scores (version 2). I made annotation databases for all mutations with GERP++>2 in human genome, this threshold is typically regarded as evolutioanrily conserved and potentially functional. Anything less than 2 is not informative, which helps reduce file size substantially. | NA | ANNOVAR annotation |
| LJB2_PhyloP | whole-exome PhyloP scores (version 2). PhyloP score is based on multiple alignments of 46 genomes. The larger the score, the more conserved the site. | NA | ANNOVAR annotation |
| LJB2_SiPhy | whole-exome SiPhy scores. SiPhy score is based on 29 mammalian genomes. The larger the score, the more conserved the site. | NA | ANNOVAR annotation |

| *FILTER, InbreedingCoeff, and DS annotations are not applicable for TSO NGS sequencing experiments. |
| --- |

**Panel validation using Coriell samples**

DNA from the following genotype-known individuals from the Coriell Cell Repositories (Coriell Institute for Medical Research, Camden, NJ, USA) was obtained and sequenced to test and validate our disease-targeted panel and our bioinformatics pipeline: NA12878, NA19240, NA12003, NA19449, NA19982, NA19704 and NA11931. Independent genotypic information for the well-studied NA12878 (sequenced 3 times for inter-run variability), NA19240, and NA12003, NA19449, NA19982, NA19704 and NA11931 were obtained from independently generated available sources [13-15]. Variants found in these samples were classified as either true positive (TP, SNPs detected in our NGS experiment that matched the independent genotypic information), false negative (FN, SNPs not detected in our NGS experiment that were present in the independent genotypic information), or false positive (FP, SNPs detected in our NGS experiment that that were absent in the independent genotypic information).

**Post-bioinformatics filtering of variants in pacemaker cohort**

After sequencing of the 9 pacemaker individuals using our custom HaloPlex pan-cardio panel, variants detected by the bioinformatics pipeline were categorized as either found or not found in the HGMD. Variants found in the HGMD database were initially classified as described in Table H. Variants that were located within *TNXB* and *ADAMTSL2* and within regions of known segmental duplications were excluded for potentially being FP variants. Segmental Duplications (or genomic duplications) are defined as regions of 1kb or more that have at least 90% similarity with another region of the genome (and at least 500 bp of non-RepeatMasked sequence) [16, 17]. Due to the size of these repeats, it is likely that our sequencing approach of 2x150 bp is not be able to differentiate between the targeted and the duplicated regions. Further variant classification following literature analyses is described in the main text. Classification of variants not found in the HGMD is described in the main text.

**Table H: HGMD initial variant classification.**

| Variant classification | HGMD_Variant_ Class (see Table G) | Additional requirements |
| --- | --- | --- |
| Affects function in isolation | DM | ESP and 1000G ≤ 5% |
| May or not affect function in isolation | DM? or DFP | ESP and 1000G ≤ 5% |
| Affects function as a modifier | DM, DM?, or DFP | ESP or 1000G > 5% but < 50% |
| Variant of unknown significance (VUS) | FTV, DP, FP | ESP and 1000G ≤ 5% |
| Likely does not affect function/does not affect function | DM, DM?, or DFP  FTV, DP, FP | ESP or 1000G > 50%  ESP or 1000G > 5% |

**Big Dye Sanger sequence confirmation of selected variants found by NGS**

Polymerase chain reaction (PCR) for selected variants was performed using HotStar Taq DNA Polymerase kit (Qiagen, Germantown, MD), except for KCNH2-10bF and KCNH2-10bR primer reaction (see below). Briefly, 50 ng of genomic DNA was amplified in a 25 µL reaction that contained 1 µM of each M13-tailed primer (Integrated DNA Technologies, Inc., Coralville, IA; Table I), 0.2 mM GeneAmp dNTP blend (Life Technologies, Carlsbad, CA), 1 mM MgCl^2+^, 1 unit of HotstarTaq DNA polymerase, 10x buffer with 15mM MgCl^2+^ and 5x Q-solution. Touchdown PCR was performed using the following reaction conditions: 95°C for 15 min; 14 cycles of 94°C for 45 sec, 65°C for 30 sec with a 0.5°C decrease per cycle, and 72°C for 1 min; 24 cycles of 94°C for 45 sec, 58°C for 30 sec, and 72°C for 1 min; 72°C for 10 min. PCR products were analyzed using agarose gel electrophoresis to confirm appropriate amplification. Products were then sequenced by automatic fluorescent DNA sequencing using an Applied Biosystems (ABI) Prism 3130*xl* Genetic Analyzer in conjunction with the ABI BigDye Terminator v3.1 cycle sequencing kit chemistry and protocol (ABI, Foster City, CA). Sequences were analyzed using Mutation Surveyor software V4.0.7 (SoftGenetics, State College, PA).

**Table I: Primers for Sanger/Big Dye variant confirmation.**

| **Patient ID** | **Gene** | **Variant (cDNA and protein)** | **Primer pair and sequence** |
| --- | --- | --- | --- |
| 9 | *ABCA1* | c.5383-3insTT | ABCA1-41F: TGTAAAACGACGGCCAGTTGACCCAGACATGAGCAATT |
|  |  |  | ABCA1-41R: CAGGAAACAGCTATGACCCCTGTGCTTAGTCACCTGCT |
| 6 | *ABCC9* | c.2200G>A (p.V734I) | ABCC9-17F: TGTAAAACGACGGCCAGTCCATTTGGGAAATGTGCTCT |
|  |  |  | ABCC9-17R: CAGGAAACAGCTATGACCGTTGCGAACCACTGCTGTT |
| 9 | *ACADL* | c.932G>T (p.R311M) | ACADL-8-2F: TGTAAAACGACGGCCAGTACTCCATTTTTGGCAACAGA |
|  |  |  | ACADL-8-2R: CAGGAAACAGCTATGACCCCTTAGTGATCTCATCCACTTCC |
| 6 | *AGL* | c.4162-1G>A | AGL-31F: TGTAAAACGACGGCCAGTTCTACGGCCAAAAACAGTCC |
|  |  |  | AGL-31R: CAGGAAACAGCTATGACCATGGCATCTCCTTTTGTTGC |
| 3, 8 | *ALMS1* | c.75_80delAGAGGA (p.26_27delEE) | ALMS1-1F: TGTAAAACGACGGCCAGTCTAAGCTGGGCCACAACC |
|  |  |  | ALMS1-1R: CAGGAAACAGCTATGACCGAGTCTGGGCCGCCTACTA |
| 7 | *APOB* | c.10580G>A (p.R3527Q) | APOB-26*F: TGTAAAACGACGGCCAGTGCTTAGCTTGGAAAGCCTCA |
|  |  |  | APOB-26*R: CAGGAAACAGCTATGACCCTGTGCTCCCAGAGGGAATA |
| 1 | *CACNB2* | c.1965T>G (p.D655E) | CACNB2-14bF: TGTAAAACGACGGCCAGTCACCACAACCATCGCAGTG |
|  |  |  | CACNB2-14bR: CAGGAAACAGCTATGACCAGGACTGCAGCCATACCAG |
| 2, 5,6 | *CALM1* | c.-218C>T | CALM1-5'UTRF: TGTAAAACGACGGCCAGTCACACCTGACCCGAGACC |
|  |  |  | CALM1-5'UTRR: CAGGAAACAGCTATGACCGAAGAAATCTCGGGCAGCTG |
| 1 | *COL1A2* | c.2123G>A (p.R708Q) | COL1A2-35/36/37F: TGTAAAACGACGGCCAGTCCACCACTGTTCTCTCTCCC |
|  |  |  | COL1A2-35/36/37R: CAGGAAACAGCTATGACCTGGTATATTCCCTGTTGCATAGC |
| 7 | *COL3A1* | c.3938A>G (p.K1313R) | COL3A1-49F: TGTAAAACGACGGCCAGTTGCAGACACATTAGCAGTCA |
|  |  |  | COL3A1-49R: CAGGAAACAGCTATGACCTGTACCAACCTAGTAACTTTGCT |
| 4, 7 | *DSG2* | c.877A>G (p.I293V) | DSG2-8F: TGTAAAACGACGGCCAGTCAAGAGATGGCAATGGAGAAGT |
|  |  |  | DSG2-8R: CAGGAAACAGCTATGACCCTTTTAAGTGTTCAGGGCTCAAA |
| 8 | **DSP* | c.29G>A (p.R10Q) | DSP-1F: TGTAAAACGACGGCCAGTCCGTCCGCCTATCCTTGG |
|  |  |  | DSP-1R: CAGGAAACAGCTATGACCAAGTTCTTTCGGGACCTGGG |
| 3 | **GATA4* | c.229G>C (p.G77R) | GATA4-2F: TGTAAAACGACGGCCAGTGAGAGAGGACACCGAAGCC |
|  |  |  | GATA4-2R: CAGGAAACAGCTATGACCCTCGCGCTCCTACTCACC |
| 8 | *GLA* | c.-30G>A | GLA-1F: TGTAAAACGACGGCCAGTTGGAAATAGGGCGGGTCAAT |
|  |  |  | GLA-1R: CAGGAAACAGCTATGACCGTTCCCGTTGAGACTCTCCA |
| 3 | *KCNH2* | c.2503delC (p.L835Cfs) | KCNH2-10bF: TGTAAAACGACGGCCAGTCTGGACTGGAAATGCCCTCT |
|  |  |  | KCNH2-10bR: CAGGAAACAGCTATGACCTTGCTTTGGATGTGTCAAGG |
| 9 | **KCNH2* | c.845C>T (p.A282V) | KCNH2-4bF: TGTAAAACGACGGCCAGTGGTGGACGTGGACCTGAC |
|  |  |  | KCNH2-4bR: CAGGAAACAGCTATGACCAGCGCAACAAGCCACTTAAT |
| 4 | *LDB3* | c.349G>A (p.D117N) | LDB3-5F: TGTAAAACGACGGCCAGTGATCTCTCTCGACACCCACC |
|  |  |  | LDB3-5R: CAGGAAACAGCTATGACCCTGAAGTCACTGCCTTGGG |
| 5 | *MEF2A* | c.1289_1291delACG (p.430delQ) | MEF2A-12F: TGTAAAACGACGGCCAGTAGACTCTGGGCCCTTTTCC |
|  |  |  | MEF2A-12R: CAGGAAACAGCTATGACCACTCATGTCCTTATTTACCGATT |
| 6 | *MIB1* | c.293G>A (p.W98X) | MIB1-2F: TGTAAAACGACGGCCAGTGTTGAGATCCATTATCTTGAAGC |
|  |  |  | MIB1-2R: CAGGAAACAGCTATGACCATATGGCAGATTTGGTCCAC |
| 7 | *MYH7* | c.3337-4_3337-3insG | MYH7-27F: TGTAAAACGACGGCCAGTTGGAAGAGCTAAACTGACTTG |
|  |  |  | MYH7-27R: CAGGAAACAGCTATGACCCATGAAGGAAGAGACACTACA |
| 7 | *SCN5A* | c.659C>T (p.T220I) | SCN5A-5F: TGTAAAACGACGGCCAGTCAAGGGAGAGGGCTGGTC |
|  |  |  | SCN5A-5R: CAGGAAACAGCTATGACCCTTCTGGAGGGTTGCCTTG |
| 7 | *SHOC2* | c.980T>G (p.L327X) | SHOC2-5F: TGTAAAACGACGGCCAGTGCATTTGTGTTGGGACTGCT |
|  |  |  | SHOC2-5R: CAGGAAACAGCTATGACCGTACCCACTCCCCACACAAT |
| 3 | *TRDN* | c.889delT (p.S297fs) | TRDN-9F: TGTAAAACGACGGCCAGTCTTCTGGAAGCTGAGGGTCA |
|  |  |  | TRDN-9R: CAGGAAACAGCTATGACCAGGGGACAGATCTAGCTTTT |
| 8 | *TRIM63* | c.143C>T (p.A48V) | TRIM63-1F: TGTAAAACGACGGCCAGTGGGCTCATGTGACCAAGATC |
|  |  |  | TRIM63-1R: CAGGAAACAGCTATGACCTGCAGGGGTTCACTTCCAA |
| 4, 6 | *TRPM4* | c.2531G>A (p.G844D) | TRPM-4F: TGTAAAACGACGGCCAGTCTGTGTGCCCCGCTCC |
|  |  |  | TRPM-4R: CAGGAAACAGCTATGACCCCAGCACTGACACCACCA |

* False Positive (FP) by Sanger/Big Dye confirmation;

Underlined: M13F/M13R primer sequence.

For the KCNH2-10bF and KCNH2-10bR primer reaction, PCR was performed using FailSafe PCR System with PreMix (Epicentre, Madison, WI). Briefly, 50 ng of genomic DNA was amplified in a 25 µL reaction that contained 0.4 µM of each M13-tailed primer (Integrated DNA Technologies, Inc., Coralville, IA; Table I), 12.5 µL 2X FailSafe PCR mix K, and 0.625 unit of FailSafe Enzyme mix. PCR was performed using the following reaction conditions: 95°C for 10 min; 35 cycles of 94°C for 1 min, 65°C for 45 sec, and 72°C for 1 min; 72°C for 10 min. PCR products were analyzed and sequenced as described above.

**Analytic performance assessment**

Analytic performance was investigated using the Coriell and the pacemaker cohort samples. Depth of coverage metrics was measured for all data sets. The expected and obtained coverage for each target region were compared across all samples. To evaluate accuracy, sensitivity and specificity, variants for Coriell samples generated by our pipeline were compared with the variants available from secondary sources. SNP sensitivity was calculated as the number of TP calls divided by number of known SNPs in target loci from secondary data (that is, TP plus FN SNPs). SNP specificity was calculated as the number of TP calls divided by number of SNPs produced by NGS experiment (that is, TP plus FP SNPs). To assess precision (repeatability via intra-run variability), NA12878 was sequenced three times in the same MiSeq run. Overall genotype concordance (OGC), non-reference sensitivity (NRS), non-reference discrepancy (NRD) and non-reference genotype concordance (NRGC) were computed as previously published [18, 19].

**Supplemental Results**

**HaloPlex custom coverage, sequence depth, and variability comparison between Coriell and pacemaker samples sequencing runs**

Using an Illumina MiSeq to sequence all Coriell and pacemaker patient HaloPlex prepared libraries, a cluster density of 760 – 970k/mm^2^ was obtained. MiSeq sequence runs were of high quality, with an obtained 91.1% of bases above Q30 for Coriell samples sequenced, and an average of 92.6% of bases above Q30 for pacemaker patients sequenced. To evaluate the target region coverage of our custom-designed HaloPlex panel, the target loci coverage of our NGS experiments were determined. Expected and observed coverages for each target region of the samples were assessed using Pearson correlation coefficient. High correlation was observed between the expected and obtained target region coverage; the Pearson correlation coefficient was above 0.88 for Coriell and pacemaker samples (range of 0.88-0.96, average 0.923), with the Coriell samples with an average of 0.922, NA12878 (run 1, 2, 3) with an average of 0.927, and pacemaker samples with an average of 0.923 (S2 Fig). A tight correlation (≥ 0.95) was found between each pair of samples as well. This indicated we obtained consistent coverage within and between our MiSeq runs. Coverage per sample and per targeted gene for each individual was determined as shown in Table B in S2 File. Overall, there was acceptable variability in target coverage among samples and cohorts and between expected (Agilent *in silico* SureDesign web tool expected limits) and obtained coverage.

**S2 Fig. Pair-wise comparison of the coverage of 246 target genes.** Pearson correlation coefficient of the coverage of 246 target genes was calculated for each pair of the experiment. The red dots represent Pearson correlation coefficients between the expected target region coverage and the observed target region coverage of each experiment/sample; the blue dots show correlation coefficients between pairs of samples. The darker blue color indicates overlapped points, the darker the color, the more number of overlapped points.

(TIF)

The precision of the HaloPlex custom panel was calculated by running NA12878 three times (repeatability tested by intra-run variability). Several measurements were used to assess run variability (Table J). Overall genotype concordance (OGC), non-reference sensitivity (NRS), non-reference discrepancy (NRD) and non-reference genotype concordance (NRGC) were computed as previously published [18, 19]. OGC, NRS, NRD and NRGC were calculated treating each replicate alternatively as comparison set and evaluation set. OGC, NRGC, NRS, and NRD showed acceptable variability with OGC range of 0.945-0.952, NRGC range of 0.967-0.975, NRS range of 0.974-0.982, and NRD range of 0.049-0.056 (Table J).

**Table J: HaloPlex intra-run performance.**

| **Comparison set** | **Evaluation set** | **OGC^*^** | **NRGC^*^** | **NRS^*^** | **NRD^*^** |
| --- | --- | --- | --- | --- | --- |
| NA12878-1 | NA12878-2 | 0.952 | 0.967 | 0.975 | 0.049 |
| NA12878-1 | NA12878-3 | 0.952 | 0.968 | 0.978 | 0.049 |
| NA12878-2 | NA12878-1 | 0.952 | 0.975 | 0.981 | 0.049 |
| NA12878-2 | NA12878-3 | 0.945 | 0.969 | 0.975 | 0.056 |
| NA12878-3 | NA12878-1 | 0.952 | 0.972 | 0.982 | 0.049 |
| NA12878-3 | NA12878-2 | 0.945 | 0.968 | 0.974 | 0.056 |

^*^ Overall genotype concordance (OGC), non-reference sensitivity (NRS), non-reference discrepancy (NRD) and non-reference genotype concordance (NRGC) were computed as previously published.^12, 13^

In general, it is expected that higher sequence depth should correlate with reliability in variant calling, while low sequence depth may result in high occurrence of false negative (FN) and false positive (FP) calls, as well as improper assignment of allelic states [20]. The overall target region average sequence depth and the exonic and splicing only (focus regions of our analyses) sequence depth were calculated for our experiments and resulted in an average of 217x and 223x, respectively, for Coriell samples, and an average of 194x and 200x, respectively, for pacemaker samples (Table B); these numbers are very close to the Agilent HaloPlex recommended minimum sequencing coverage depth of 200x. There was an acceptable variability in sequence depth among various sequencing runs (S3 Fig). Overall, the target enrichment was successful with less than 0.01% of aligned bases being off target (Table B). Although the variability in coverage and sequence depth between Coriell samples and pacemaker patients was acceptable, an explanation for these differences may be due to the total number of reads passing filter (PF) (the total number of passing filter reads for each sample run) from each indexed sample in each MiSeq run (Table B). It was observed that there was an intermediate linear relationship between the exonic and splicing only sequence depth and the total number of reads PF in all Coriell and pacemaker samples combined (R-square = 0.55), a weak linear relationship in Coriell samples (R-square = 0.12), and a strong linear relationship in pacemaker patients (R-square = 1) (S4 Fig). This is further supported by the fact that there was an intermediate difference in number of reads PF between Coriell samples and pacemaker patients (Table B). Additionally, variants such as DNA quality, library preparation efficiency, index selection, concentration of library added to sequencer, pipetting error, sequencer performance (indicated by the % bases ≥Q30), and bioinformatics analyses may contribute to these deviations. Of note, all Coriell DNAs were prepared and sequenced in one 11-plex experiment, whereas all pacemaker patient DNAs were prepared and sequenced in separate experiments. It is up to each laboratory to decide the acceptable range of variability, as long as the efficacy of detecting an underlying sequence change is not affected and the requirements of minimum and average sequence depth established for the specific panel are met.

**Table B: HaloPlex NGS depth of coverage for Coriell and pacemaker samples.**

| **Sample ID** | **# Reads PF*** | **% ≥ Q30^†^** | **% Off Bait^‡^** | **Target region mean depth (x)** | **Exonic/ splicing mean depth (x)** | **% target loci with depth ≥15x** | **Mean depth in target loci with depth ≥15x (x)** |
| --- | --- | --- | --- | --- | --- | --- | --- |
| NA12878-1 | 1,675,055 | 91.1 | 0.0115 | 263 | 270 | 0.95 | 275 |
| NA12878-2 | 1,168,454 | 91.1 | 0.0112 | 184 | 189 | 0.93 | 197 |
| NA12878-3 | 1,365,928 | 91.1 | 0.0111 | 213 | 219 | 0.95 | 224 |
| NA11931 | 1,159,731 | 91.1 | 0.0105 | 229 | 235 | 0.95 | 240 |
| NA12003 | 1,496,658 | 91.1 | 0.0155 | 219 | 225 | 0.95 | 231 |
| NA19240 | 1,570,562 | 91.1 | 0.0122 | 183 | 188 | 0.93 | 196 |
| NA19449 | 1,360,146 | 91.1 | 0.0162 | 230 | 236 | 0.95 | 242 |
| NA19704 | 1,436,083 | 91.1 | 0.0125 | 219 | 225 | 0.95 | 230 |
| NA19982 | 1,451,496 | 91.1 | 0.0120 | 210 | 216 | 0.95 | 222 |
| **Average Coriell** | **1,409,346** | **91.1** | **0.0125** | **216.67** | **222.56** | **0.95** | **228.56** |
| Pacemaker 1 | 966,253 | 93.0 | 0.0102 | 155 | 160 | 0.94 | 166 |
| Pacemaker 2 | 1,153,984 | 93.0 | 0.0105 | 184 | 189 | 0.94 | 195 |
| Pacemaker 3 | 1,034,588 | 93.0 | 0.0104 | 163 | 167 | 0.94 | 173 |
| Pacemaker 4 | 1,237,878 | 92.4 | 0.0098 | 197 | 203 | 0.95 | 209 |
| Pacemaker 5 | 1,196,137 | 92.4 | 0.0097 | 192 | 197 | 0.95 | 202 |
| Pacemaker 6 | 1,539,337 | 92.4 | 0.0102 | 240 | 247 | 0.96 | 250 |
| Pacemaker 7 | 1,288,083 | 92.4 | 0.0106 | 204 | 210 | 0.94 | 216 |
| Pacemaker 8 | 1,262,767 | 92.4 | 0.0096 | 203 | 208 | 0.95 | 214 |
| Pacemaker 9 | 1,335,116 | 92.4 | 0.0108 | 212 | 218 | 0.95 | 223 |
| **Average Pacemaker** | **1,223,794** | **92.6** | **0.0102** | **194.44** | **199.89** | **0.95** | **205.33** |

* Reads PF: the total number of passing filter reads per MiSeq sequencing lane

^†^ % ≥ Q30: percentage of bases with QScore higher or equal than 30 from MiSeq sequencing experiment (Q30 chance of wrong base call = 0.1%)

^‡^ % off bait: the percentage of aligned PF bases that mapped neither on nor near a bait

**S3 Fig. Percentage of target bases covered at the indicated coverage thresholds.** Each color represents results from the sequence run of a Coriell or pacemaker implanted patient.

(TIF)

**S4 Fig. Correlation of reads passing filter (PF) with obtained sequence depth.**

(TIF)

According to current American College of Medical Genetics (ACMG) clinical NGS guidelines, laboratories use 10-20x as a minimum sequence depth per target nucleotide (minimum sequence depth should be determined empirically for each NGS panel in each laboratory) [20]. For our custom HaloPlex NGS pan-cardiovascular panel, at a minimum of 15x sequence depth, an average of 95% of target regions were covered with a mean read depth of 229x for Coriell samples, and an average of 95% of target regions were covered with a mean read depth of 205x for pacemaker patients (Table B). There was a small difference in average percent of target regions covered with at least 15 fold and in mean read depth of target regions covered with at least 15 fold between Coriell samples and pacemaker patients showing that there was an acceptable variability among various sequencing runs. Again, although the variability in mean read depth of target regions covered with at least 15 fold between Coriell samples and pacemaker patients was small, an explanation for these differences may be due to the total number of reads PF from each indexed sample in each MiSeq run. It was observed that there was an intermediate linear relationship between the mean read depth of target regions covered with at least 15 fold and the total number of reads PF in all Coriell and pacemaker samples combined (R-square = 0.56), a weak linear relationship in Coriell samples (R-square = 0.12), and a strong linear relationship in pacemaker patients (R-square = 0.99) (S4 Fig).

**HaloPlex SNP performance for Coriell sequencing runs**

Other than coverage and sequence depth, the performance of SNP variant calling for genotype known Coriell individuals (from secondary available databases) was examined in order to better access both the quality of our custom HaloPlex NGS platform and of our post-sequence bioinformatics pipeline. Table K summarizes the SNP variant calling results obtained for 9 sequencing runs of Coriell individuals (NA12878 sequenced in triplicate). For the 9 Coriell sequencing runs, SNP specificity for all target regions had an inferior performance than the SNP sensitivity due to the large number of FP calls that were made in comparison to secondary available data. This difference may be due to fine-tunings of our in-house bioinformatics pipeline in attempts to minimize FN calls as much as possible, as these are not able to be confirmed in a clinical scenario of genotype unknown samples as they are inherently non-existent (other in-house pipelines had high specificity at the cost of very poor sensitivity and a large number of FN variants – data not shown). This is in contrast with FP calls, which can be confirmed by Sanger sequencing. Another observation was that a large difference in specificity was observed between the three Coriell NA12878 runs and the other six Coriell samples. This difference does not seem to correlate with the average sequence depth for the SNPs analyzed, but they may be associated with the fact that SNP secondary data for the well-studied NA12878 came from a different source. Additionally, as expected, the SNP specificity and sensitivity had a trend towards improving performances as sequence depth increased (data not shown).

**Table K: Overall Performance of SNP variant calling in genotype known Coriell samples^*^.**

| **Individual** | **Known SNPs in target loci (secondary data)** | **MiSeq SNPs in target loci** | **# TP SNPs** | **# FP SNPs** | **# FN SNPs** | **Spec.** | **Sens.** | **Mean depth for SNPs** |
| --- | --- | --- | --- | --- | --- | --- | --- | --- |
| NA12878-1 | 433 | 682 | 412 | 270 | 21 | 0.604 | 0.951 | 311.90 |
| NA12878-2 | 433 | 678 | 410 | 268 | 23 | 0.604 | 0.946 | 222.41 |
| NA12878-3 | 433 | 679 | 412 | 267 | 21 | 0.606 | 0.951 | 247.15 |
| NA19240 | 702 | 848 | 694 | 154 | 8 | 0.818 | 0.988 | 211.37 |
| NA12003 | 632 | 662 | 589 | 73 | 43 | 0.889 | 0.931 | 263.74 |
| NA19449 | 866 | 931 | 817 | 114 | 49 | 0.876 | 0.943 | 268.03 |
| NA19982 | 793 | 858 | 774 | 84 | 19 | 0.902 | 0.976 | 237.83 |
| NA19704 | 831 | 891 | 795 | 96 | 36 | 0.892 | 0.956 | 252.55 |
| NA11931 | 624 | 675 | 603 | 72 | 21 | 0.893 | 0.966 | 263.40 |

^*^ Secondary data: independent genotypic information for NA12878, NA19240, NA12003, NA19449, NA19982, NA19704 and NA11931 were obtained from independently generated available sources [13-15].

**#** = number of; **FN** = false negative; **FP** = false positive; **Sens**. = sensitivity; **SNPs** = single nucleotide polymorphisms; **Spec**. = specificity; **TP** = true positive

Poor SNP performance indicated by a large number of FN and FP calls is a severe problem in any scenario, being it in the clinical setting or in research experiments, as they may lead to failure to detect important disease causing variants and may demand expensive confirmation of a large number of variants by orthologous technologies. With that in mind, a more careful analysis of our SNP performance data was implemented. Table L summarizes a multi-layer analysis of SNP performance by selecting specific SNP characteristics that were originally desired when our custom panel was designed. Overall, the percent of FP and FN SNP calls drastically decreased as we selected SNPs within exonic and splicing regions of our target genes (that is, removed any SNPs from non-splicing intronic, UTR, and intergenic regions which were not purposely targeted and were expected to have poor performance), followed by removal of SNPs that were located within regions of segmental duplications, followed by removal of SNPs that had a lower than 15x sequence depth. Given this analysis, we are confident that our NGS platform and bioinformatics analysis pipeline can produce data with less than 8% FP calls (range of 2.33 to 7.32) and less than 5% FN calls (range of 0.63 to 4.23) for exonic and splicing regions of all 246 cardiovascular genes with a minimum of 15x sequencing depth (95% of targets, see TableB) after removal of regions of segmental duplications.

**Table L: Selective analyses of SNP performance in Coriell samples^*^.**

|  | **All target loci** | | | **Exonic and splicing loci** | | | **Exonic and splicing loci minus SegDup** | | | **Exonic and splicing loci minus SegDup; ≥15x depth only** | | |
| --- | --- | --- | --- | --- | --- | --- | --- | --- | --- | --- | --- | --- |
| **Individual** | **TP (%)** | **FP (%)** | **FN (%)** | **TP (%)** | **FP (%)** | **FN (%)** | **TP (%)** | **FP (%)** | **FN (%)** | **TP (%)** | **FP (%)** | **FN (%)** |
| NA12878-1 | 95.15 | 62.36 | 4.85 | 95.81 | 6.15 | 4.19 | 97.90 | 6.29 | 2.10 | 99.38 | 6.56 | 0.63 |
| NA12878-2 | 94.69 | 61.89 | 5.31 | 95.81 | 6.42 | 4.19 | 97.90 | 6.59 | 2.10 | 99.36 | 6.07 | 0.64 |
| NA12878-3 | 95.15 | 61.66 | 4.85 | 95.53 | 5.59 | 4.47 | 97.60 | 5.69 | 2.40 | 99.07 | 5.59 | 0.93 |
| NA19240 | 98.86 | 21.94 | 1.14 | 98.57 | 13.60 | 1.43 | 98.55 | 8.23 | 1.45 | 99.24 | 7.32 | 0.76 |
| NA12003 | 93.20 | 11.55 | 6.80 | 92.64 | 4.36 | 7.36 | 94.25 | 2.87 | 5.75 | 95.77 | 2.72 | 4.23 |
| NA19449 | 94.34 | 13.16 | 5.66 | 93.66 | 8.71 | 6.34 | 94.95 | 6.95 | 5.05 | 96.04 | 5.29 | 3.96 |
| NA19982 | 97.60 | 10.59 | 2.40 | 97.85 | 3.65 | 2.15 | 98.42 | 2.93 | 1.58 | 99.30 | 2.33 | 0.70 |
| NA19704 | 95.67 | 11.55 | 4.33 | 96.60 | 4.88 | 3.40 | 97.32 | 3.13 | 2.68 | 99.07 | 3.02 | 0.93 |
| NA11931 | 96.63 | 11.54 | 3.37 | 96.74 | 6.25 | 3.26 | 96.89 | 3.39 | 3.11 | 99.12 | 3.24 | 0.88 |
| **Average** | **95.70** | **29.58** | **4.30** | **95.91** | **6.62** | **4.09** | **97.09** | **5.12** | **2.91** | **98.48** | **4.68** | **1.52** |

^*^ Secondary data: independent genotypic information for NA12878, NA19240, NA12003, NA19449, NA19982, NA19704 and NA11931 were obtained from independently generated available sources [13-15].

**FN** = false negative; **FP** = false positive; **SegDup** = segmental duplications (or genomic duplications); **TP** = true positive

**Supplemental References**

1. Crotti L, Lewandowska MA, Schwartz PJ, Insolia R, Pedrazzini M, Bussani E, et al. A KCNH2 branch point mutation causing aberrant splicing contributes to an explanation of genotype-negative long QT syndrome. Heart Rhythm. 2009; 6(2):212-218.
2. Agilent SureDesign web tool. Available: https://earray.chem.agilent.com/suredesign/.
3. Li H, Durbin R. Fast and accurate short read alignment with Burrows-Wheeler transform. Bioinformatics. 2009; 25(14):1754-1760.
4. Li H, Handsaker B, Wysoker A, Fennell T, Ruan J, Homer N, et al. The Sequence Alignment/Map format and SAMtools. Bioinformatics. 2009; 25(16):2078-2079.
5. McKenna A, Hanna M, Banks E, Sivachenko A, Cibulskis K, Kernytsky A, et al. The Genome Analysis Toolkit: a MapReduce framework for analyzing next-generation DNA sequencing data. Genome Res. 2010; 20(9):1297-1303.
6. ANNOVAR. Available: http://www.openbioinformatics.org/annovar/annovar_download.html.
7. Human Gene Mutation Database. HGMD Professional 2014.2, BIOBASE, Beverly, MA. Available: http://www.hgmd.org/.
8. Wang K, Li M, Hakonarson H. ANNOVAR: functional annotation of genetic variants from high-throughput sequencing data. Nucleic Acids Res. 2010; 38(16):e164.
9. Stenson PD, Mort M, Ball EV, Howells K, Phillips AD, Thomas NS, et al. The Human Gene Mutation Database: 2008 update. Genome Med. 2009; 1(1):13.
10. Stenson PD, Mort M, Ball EV, Shaw K, Phillips A, Cooper DN. The Human Gene Mutation Database: building a comprehensive mutation repository for clinical and molecular genetics, diagnostic testing and personalized genomic medicine. Hum Genet. 2014; 133(1):1-9.
11. R Development Core Team. R: a language and environment for statistical computing. Vienna, Austria, R Foundation for Statistical Computing.
12. Wickham H. ggplot2: elegant graphics for data analysis. 2nd ed. Springer New York; 2009.
13. NA12878 genetic information. Available: ftp://ftp.platinumgenomes.org/trio.
14. NA19240 genetic information. Available: ftp://ftp-trace.ncbi.nih.gov/1000genomes/ftp/pilot_data/release/2010_07/trio/snps/.
15. NA12003, NA19449, NA19982, NA19704 and NA11931 genetic information from the 1000 genomes project. Available: ftp://ftp-trace.ncbi.nih.gov/1000genomes/ftp/release/20110521/.
16. Bailey JA, Gu Z, Clark RA, Reinert K, Samonte RV, Schwartz S, et al. Recent segmental duplications in the human genome. Science. 2002; 297(5583):1003-1007.
17. Bailey JA, Yavor AM, Massa HF, Trask BJ, Eichler EE. Segmental duplications: organization and impact within the current human genome project assembly. Genome Res. 2001; 11(6):1005-1017.
18. Linderman MD, Brandt T, Edelmann L, Jabado O, Kasai Y, Kornreich R, et al. Analytical validation of whole exome and whole genome sequencing for clinical applications. BMC Med Genomics. 2014; 7:20.
19. DePristo MA, Banks E, Poplin R, Garimella KV, Maguire JR, Hartl C, et al. A framework for variation discovery and genotyping using next-generation DNA sequencing data. Nat Genet. 2011; 43(5):491-498.
20. Rehm HL, Bale SJ, Bayrak-Toydemir P, Berg JS, Brown KK, Deignan JL, et al. ACMG clinical laboratory standards for next-generation sequencing. Genet Med. 2013; 15(9):733-747.
21. Shihab HA, Gough J, Cooper DN, Stenson PD, Barker GL, Edwards KJ, et al. Predicting the functional, molecular, and phenotypic consequences of amino acid substitutions using hidden Markov models. Hum Mutat. 2013; 34(1):57-65.

**Table A: Exclusion criteria for genetic testing in patients with a first degree relative with a pacemaker.**

| Reason for exclusion * | Number of patients |
| --- | --- |
| ≥ Moderate valvular disease | 5 |
| Left ventricular hypertrophy | 5 |
| Cardiomyopathy | 4 |
| Coronary artery disease | 4 |
| Post-operative ABV† | 3 |
| AVB in the setting of subarachnoid hemorrhage | 1 |
| Carotid hypersensitivity | 1 |
| Insufficient records | 1 |

* Some patients had more than one reason for exclusion.

†Including post electrophysiology ablation.

**AVB** = atrioventricular block.

**Table C: Selected HGMD and nonHGMD VUSs in pacemaker patients.**

| **ID** | **CHR:POS** | **rsID** | **GT** | **GENE** | **Type** | **Transcript** | **Variant (cDNA and protein)** |
| --- | --- | --- | --- | --- | --- | --- | --- |
| 1 | 18:29122618 | rs79241126 | het | *DSG2* | NSN | NM_001943 | **c.2137G>A (p.E713K)** |
| 1 | 17:78092585 | rs1800315 | het | *GAA* | NSN | NM_001079804 | c.2780C>T (p.T927I) |
| 1 | X:108867996 | N/A | hemi | *KCNE1L* | NSN | NM_012282 | **c.254G>A (p.R85H)** |
| 1 | 19:11221457 | rs12710260 | het | *LDLR* | In | NM_000527 | **c.1060+10G>C** |
| 1 | 9:139401233 | rs61751543 | het | *NOTCH1* | NSN | NM_017617 | **c.3836G>A (p.R1279H)** |
| 1 | 5:155935708 | rs45559835 | het | *SGCD* | NSN | NM_001128209 | c.287G>A (p.R96Q) |
| 1 | 10:75843100 | rs1908339 | homo | *VCL* | In | NM_014000 | **c.875-24T>A** |
| 2 | 18:29101156 | rs191143292 | het | *DSG2* | NSN | NM_001943 | **c.473T>G (p.V158G)** |
| 2 | 6:133789765 | rs41286200 | het | *EYA4* | NSN | NM_172105 | c.866C>T (p.T289M) |
| 2 | 16:86613301 | N/A | het | *FOXL1* | NFS del | NM_005250 | c.973_987del (p.325_329delLGIPF) |
| 2 | 19:11221457 | rs12710260 | het | *LDLR* | In | NM_000527 | **c.1060+10G>C** |
| 2 | 17:16203290 | rs115958467 | het | *PIGL* | NSN | NM_004278 | c.424C>A (p.L142M) |
| 2 | 1:237664100 | N/A | het | *RYR2* | NSN | NM_001035 | c.2293A>C (p.S765R) |
| 2 | 6:152658062 | rs117501809 | het | *SYNE1* | NSN | NM_182961 | c.12442G>C (p.D4148H) |
| 2 | 6:152668353 | N/A | het | *SYNE1* | NSN | NM_182961 | c.11919T>G (p.I3973M) |
| 2 | 19:49671214 | rs144781529 | het | *TRPM4* | NSN | NM_017636 | c.308A>G (p.Y103C) |
| 2 | 2:179542468 | N/A | het | *TTN* | NFS del | NM_001256850 | c.33199_33219del (p.11067_11073delEEEEPLV) |
| 2 | 10:75843100 | rs1908339 | het | *VCL* | In | NM_014000 | **c.875-24T>A** |
| 3 | 5:178555035 | N/A | het | *ADAMTS2* | NFS ins | NM_014244 | c.2541_2542insAAC (p.V848delinsNV) |
| 3 | 2:73613035 | N/A | het | *ALMS1* | NFS del | NM_015120 | c.75_80delAGAGGA (p.26_27delEE) |
| 3 | 2:21225597 | rs61743299 | het | *APOB* | NSN | NM_000384 | **c.12697T>A (p.S4233T)** |
| 3 | 2:21229860 | rs12720855 | het | *APOB* | NSN | NM_000384 | **c.9880T>C (p.S3294P)** |
| 3 | 2:21232373 | rs12713675 | het | *APOB* | NSN | NM_000384 | **c.7367C>A (p.A2456D)** |
| 3 | 9:141016441 | rs150679456 | het | *CACNA1B* | NSN | NM_000718 | c.7010A>G (p.H2337R) |
| 3 | 8:61765273 | rs3763592 | het | *CHD7* | In | NM_017780 | **c.6103+8C>T** |
| 3 | 1:53668063 | rs75939866 | het | *CPT2* | NSN | NM_000098 | **c.302C>T (p.A101V)** |
| 3 | 3:9985673 | rs73118372 | het | *CRELD1* | NSN | NM_001031717 | **c.1136T>C (p.M379T)** |
| 3 | 9:108370120 | rs116105846 | het | *FKTN* | NSN | NM_006731 | c.668C>T (p.T223I) |
| 3 | 7:150647150 | N/A | het | *KCNH2* | FS del | NM_172056 | c.2503delC (p.L835Cfs) |
| 3 | 19:11221411 | rs139361635 | het | *LDLR* | NSN | NM_000527 | **c.1024G>A (p.D342N)** |
| 3 | 11:47359046 | rs3729952 | het | *MYBPC3* | NSN | NM_000256 | **c.2498C>T (p.A833V)** |
| 3 | 6:123786033 | rs201431159 | het | *TRDN* | FS del | NM_001256020 | c.889delT (p.S297fs) |
| 3 | 10:75843100 | rs1908339 | homo | *VCL* | In | NM_014000 | **c.875-24T>A** |
| 4 | 4:114286207 | rs66785829 | het | *ANK2* | NSN | NM_001148 | **c.10901T>A (p.V3634D)** |
| 4 | 2:21224853 | rs1801695 | het | *APOB* | NSN | NM_000384 | **c.13441G>A (p.A4481T)** |
| 4 | 18:29104714 | rs2230234 | het | *DSG2* | NSN | NM_001943 | **c.877A>G (p.I293V)** |
| 4 | 18:29122618 | rs79241126 | het | *DSG2* | NSN | NM_001943 | **c.2137G>A (p.E713K)** |
| 4 | 17:39925713 | rs41283425 | het | *JUP* | NSN | NM_002230 | c.425G>A (p.R142H) |
| 4 | 19:11221457 | rs12710260 | het | *LDLR* | In | NM_000527 | **c.1060+10G>C** |
| 4 | 17:12656546 | rs28730825 | het | *MYOCD* | NSN | NM_001146312 | c.1941G>C (p.Q647H) |
| 4 | 5:172660004 | rs72554028 | het | *NKX2-5* | SYN | NM_004387 | **c.543G>A (p.Q181Q)** |
| 4 | 19:35524944 | rs67486287 | het | *SCN1B* | NSN | NM_199037 | **c.749G>C (p.R250T)** |
| 5 | 4:114282005 | rs180843436 | het | *ANK2* | NSN | NM_001148 | **c.10708G>A (p.E3570K)** |
| 5 | 6:7581032 | rs28763967 | het | *DSP* | NSN | NM_004415 | c.4609C>T (p.R1537C) |
| 5 | 19:11221457 | rs12710260 | het | *LDLR* | In | NM_000527 | **c.1060+10G>C** |
| 5 | 15:100252713 | N/A | het | *MEF2A* | NFS del | NM_005587 | c.1289_1291delACG (p.430delQ) |
| 5 | 11:47370041 | rs3729989 | het | *MYBPC3* | NSN | NM_000256 | **c.706A>G (p.S236G)** |
| 5 | 9:128678097 | rs145687528 | het | *PBX3* | NSN | NM_006195 | **c.407C>T (p.A136V)** |
| 5 | 17:16203290 | rs115958467 | het | *PIGL* | NSN | NM_004278 | c.424C>A (p.L142M) |
| 5 | 19:35524944 | rs67486287 | het | *SCN1B* | NSN | NM_199037 | **c.749G>C (p.R250T)** |
| 5 | 1:26384907 | rs61749355 | het | *TRIM63* | NSN | NM_032588 | c.805G>A (p.E269K) |
| 5 | 10:75843100 | rs1908339 | homo | *VCL* | In | NM_014000 | **c.875-24T>A** |
| 6 | 1:100380944 | N/A | het | *AGL* | Splic | NM_000642 | c.4162-1G>A |
| 6 | 2:21249716 | rs12691202 | het | *APOB* | NSN | NM_000384 | **c.2188G>A (p.V730I)** |
| 6 | 10:18828181 | rs143326262 | het | *CACNB2* | NSN | NM_201596 | **c.1511C>T (p.T504I)** |
| 6 | 16:57015091 | rs5880 | het | *CETP* | NSN | NM_000078 | **c.1168G>C (p.A390P)** |
| 6 | 16:57017319 | rs1800777 | het | *CETP* | NSN | NM_000078 | **c.1403G>A (p.R468Q)** |
| 6 | 15:48748913 | rs140649 | het | *FBN1* | SYN | NM_000138 | **c.5343G>A (p.V1781V)** |
| 6 | X:153599549 | N/A | hemi | *FLNA* | NFS ins | NM_001110556 | c.64_65insTCG (p.D22delinsVD) |
| 6 | 17:39925713 | rs41283425 | het | *JUP* | NSN | NM_002230 | c.425G>A (p.R142H) |
| 6 | 10:88477818 | N/A | het | *LDB3* | NSN | NM_007078 | c.1774G>C (p.E592Q) |
| 6 | 19:11221457 | rs12710260 | homo | *LDLR* | In | NM_000527 | **c.1060+10G>C** |
| 6 | 18:19345796 | N/A | het | *MIB1* | Stop Gain | NM_020774 | c.293G>A (p.W98X) |
| 6 | 1:120478125 | rs147223770 | het | *NOTCH2* | NSN | NM_024408 | c.3625T>G (p.F1209V) |
| 7 | 2:73675690 | rs199573929 | het | *ALMS1* | NSN | NM_015120 | c.2033A>G (p.Y678C) |
| 7 | 2:21232044 | rs1801696 | het | *APOB* | NSN | NM_000384 | **c.7696G>A (p.E2566K)** |
| 7 | 2:189875018 | rs111840783 | het | *COL3A1* | NSN | NM_000090 | **c.3938A>G (p.K1313R)** |
| 7 | 1:53676980 | rs17848485 | het | *CPT2* | NSN | NM_000098 | **c.1634A>C (p.E545A)** |
| 7 | 18:29104714 | rs2230234 | het | *DSG2* | NSN | NM_001943 | **c.877A>G (p.I293V)** |
| 7 | 17:39925713 | rs41283425 | het | *JUP* | NSN | NM_002230 | c.425G>A (p.R142H) |
| 7 | 19:11221457 | rs12710260 | het | *LDLR* | In | NM_000527 | **c.1060+10G>C** |
| 7 | 9:103348329 | N/A | het | *MURC* | NFS del | NM_001018116 | c.692_712del (p.231_238del) |
| 7 | 11:47370041 | rs3729989 | het | *MYBPC3* | NSN | NM_000256 | **c.706A>G (p.S236G)** |
| 7 | 1:237955364 | rs114289907 | het | *RYR2* | In | NM_001035 | **c.13564-41A>G** |
| 7 | 10:112764371 | N/A | het | *SHOC2* | Stop Gain | NM_007373 | c.980T>G (p.L327X) |
| 7 | 10:75843100 | rs1908339 | homo | *VCL* | In | NM_014000 | **c.875-24T>A** |
| 8 | 1:100343254 | rs141043166 | het | *AGL* | NSN | NM_000642 | **c.1481G>A (p.R494H)** |
| 8 | 2:73613035 | N/A | het | *ALMS1* | NFS del | NM_015120 | c.75_80delAGAGGA (p.26_27delEE) |
| 8 | 16:57015091 | rs5880 | het | *CETP* | NSN | NM_000078 | **c.1168G>C (p.A390P)** |
| 8 | 16:3831230 | rs61753381 | het | *CREBBP* | NSN | NM_004380 | c.1651C>A (p.L551I) |
| 8 | 18:28648084 | rs141873745 | het | *DSC2* | NSN | NM_024422 | c.2603C>T (p.S868F) |
| 8 | 8:11614483 | rs115372595 | het | *GATA4* | NSN | NM_002052 | **c.1037C>T (p.A346V)** |
| 8 | X:100662921 | rs3027584 | het | GLA | UTR5 | NM_000169 | **c.-30G>A** |
| 8 | 19:11221457 | rs12710260 | het | *LDLR* | In | NM_000527 | **c.1060+10G>C** |
| 8 | 11:47357416 | rs3729936 | het | *MYBPC3* | In | NM_000256 | **c.2737+12G>A** |
| 8 | 14:23876267 | rs28711516 | het | *MYH6* | NSN | NM_002471 | **c.166G>A (p.G56R)** |
| 8 | 19:35524944 | rs67486287 | het | *SCN1B* | NSN | NM_199037 | **c.749G>C (p.R250T)** |
| 9 | 12:21995312 | rs147895473 | het | *ABCC9* | NSN | NM_005691 | **c.3409G>A (p.V1137I)** |
| 9 | 2:211068111 | N/A | het | *ACADL* | FS del | NM_001608 | c.928_933delinsCATGAATGTTATGTTT (p.T310fs) |
| 9 | 2:189918622 | rs116298748 | het | *COL5A2* | NSN | NM_000393 | c.2498C>T (p.P833L) |
| 9 | 2:189931144 | rs35852101 | het | *COL5A2* | NSN | NM_000393 | c.1535T>C (p.V512A) |
| 9 | 10:68526108 | rs115276158 | het | *CTNNA3* | NSN | NM_013266 | c.1195C>A (p.L399I) |
| 9 | 9:130616621 | rs35400405 | het | *ENG* | NSN | NM_001114753 | **c.14C>T (p.T5M)** |
| 9 | 6:133789804 | rs75133151 | het | *EYA4* | NSN | NM_172105 | c.905G>A (p.G302D) |
| 9 | 15:48722907 | rs363835 | het | *FBN1* | NSN | NM_000138 | **c.6832C>T (p.P2278S)** |
| 9 | 8:145700346 | rs112028242 | het | *FOXH1* | NSN | NM_003923 | c.373A>T (p.T125S) |
| 9 | 2:26432662 | rs2229420 | het | *HADHA* | NSN | NM_000182 | **c.1072C>A (p.Q358K)** |
| 9 | 19:11221357 | rs72658860 | het | *LDLR* | NSN | NM_000527 | **c.970G>A (p.G324S)** |
| 9 | 11:47371449 | rs201012766 | het | *MYBPC3* | NSN | NM_000256 | **c.530G>A (p.R177H)** |
| 9 | 19:1395487 | rs3065757 | het | *NDUFS7* | NFS ins | NM_024407 | c.642_643insCGC (p.X214delinsX) |
| 9 | 1:12009911 | rs34878020 | het | *PLOD1* | NSN | NM_000302 | c.250G>A (p.A84T) |
| 9 | 19:35524944 | rs67486287 | het | *SCN1B* | NSN | NM_199037 | **c.749G>C (p.R250T)** |
| 9 | 3:38646313 | rs199473116 | het | *SCN5A* | NSN | NM_198056 | c.1425A>C (p.R475S) |
| 9 | 17:37822049 | rs45458802 | het | *TCAP* | NSN | NM_003673 | c.191C>T (p.S64L) |
| 9 | 10:75843100 | rs1908339 | homo | *VCL* | In | NM_014000 | **c.875-24T>A** |

*Variant is likely pathogenic as autosomal recessive disease; however, the patient in question is heterozygous for the variant.

Underlined: arrhythmia associated gene

**Bold:** variants that are found in HGMD

**chr** = chromosome; **del** = deletion; **FS** = frameshift; **GT** = genotype; **hemi** = hemizygous; **het** = heterozygous; **homo** = homozygous; **ID** = Pacemaker patient identification; **in** = intronic; **ins** = insertion;

**N/A** = not applicable or not available; **NFS** = non-frameshift; **NSN** = nonsynonymous; **pos** = position; **splic** = splicing; **SYN** = synonymous; **UTR5** = variant in 5’untranslated region

**Table D: Pacemaker variants filtering analysis and classification (number of variants).**

| **Patient ID** | **1** | **2** | **3** | **4** | **5** | **6** | **7** | **8** | **9** | **total** |
| --- | --- | --- | --- | --- | --- | --- | --- | --- | --- | --- |
| NonHGMD VUS^†^ | 2 (0) | 8 (5) | 6 (1) | 2 (1) | 4 (1) | 6 (1) | 4 (1) | 3 (1) | 10 (2) | 45 (13) |
| HGMD^‡^ changes | 43 (11) | 38 (10) | 37 (8) | 46 (18) | 36 (11) | 46 (16) | 39 (9) | 42 (8) | 47 (13) | 374 (104) |
| 1.1^‡^ (if HGMD class correct) | 1 (0) | 2 (1) | 2 (0) | 4 (3) | 1 (1) | 2 (1) | 3 (1) | 3 (1) | 5 (1) | 23 (9) |
| 1.2^‡^ (if HGMD class correct) | 3 (1) | 0 (0) | 5 (0) | 0 (0) | 0 (0) | 3 (2) | 3 (1) | 1 (0) | 3 (0) | 18 (4) |
| 1.3^‡^ (if HGMD class correct) | 10 (3) | 8 (2) | 4 (1) | 11 (6) | 8 (4) | 11 (4) | 7 (1) | 9 (2) | 8 (2) | 76 (25) |
| 2^‡^ (if HGMD class correct) | 0 (0) | 0 (0) | 0 (0) | 1 (0) | 2 (0) | 2 (0) | 0 (0) | 3 (0) | 0 (0) | 8 (0) |
| 3^‡^ (if HGMD class correct) | 29 (7) | 28 (7) | 26 (7) | 30 (9) | 25 (6) | 28 (9) | 26 (6) | 26 (5) | 31 (10) | 249 (66) |
| Re-classified HGMD 1.1^‡^ | 0 (0) | 0 (0) | 1* (0) | 1 (0) | 0 (0) | 0 (0) | 0 (0) | 0 (0) | 2^§^ (0) | 4 (0) |
| Re-classified HGMD 1.2^‡^ | 1 (0) | 0 (0) | 0 (0) | 1 (1) | 0 (0) | 2 (2) | 2 (1) | 2 (0) | 0 (0) | 8 (4) |
| Re-classified HGMD 1.3^‡^ | 7 (3) | 3 (2) | 3 (2) | 4 (2) | 6 (4) | 9* (5) | 3 (1) | 4* (2) | 5 (3) | 44 (24) |
| Re-classified HGMD 2^‡^ | 5 (2) | 3 (1) | 9 (0) | 7 (5) | 6 (2) | 6 (1) | 8 (2) | 7 (2) | 8* (2) | 59 (17) |
| Re-classified HGMD 3^‡^ | 30 (6) | 32 (7) | 24 (6) | 33 (10) | 24 (5) | 29 (8) | 26 (5) | 29 (4) | 32 (8) | 259 (59) |

* One variant is associated with autosomal recessive disease; however, the patient in question is heterozygous for the variant.

^†^ Numbers reflect non-recurrent and recurrent variants, removed variants not confirmed by Sanger sequencing. Removed *TNXB* and *ADAMTSL2* variants.

^‡^ Numbers reflect recurrent and non-recurrent HGMD variants. Removed variants possibly in pseudogenes.

^§^ Two variants are associated with autosomal recessive disease; however, the patients in question are heterozygous for the variants.

(in parenthesis) = how many of the total number are in arrhythmia genes.

**1.1** = variant affects function in isolation; **1.2** = variant may or may not affect function in isolation; **1.3** = variant affects function as a modifier; **2** = VUS; **3** = variant likely does not affect function/does not affect function; **VUS** = variants of unknown significance

**Table E: HGMD variants with disease association in pacemaker patients.**

| **ID** | **Cat^†^** | **CHR:POS** | **rsID** | **GT** | **GENE** | **Type** | **Transcript** | **Variant (cDNA and protein)** |
| --- | --- | --- | --- | --- | --- | --- | --- | --- |
| 1 | 1.3 | 1: 76227022 | rs1061337 | het | ACADM | SYN | NM_001127328 | c.1173A>G (p.V391V) |
| 1 | 1.3 | 4:114278277 | rs3733617 | het | ANK2 | NSN | NM_001148 | c.8503C>T (p.P2835S) |
| 1 | 1.3 | 10:18828635 | rs58225473 | het | CACNB2 | NSN | NM_201596 | c.1965T>G (p.D655E) |
| 1 | 1.2 | 7:94049588 | rs72658163 | het | COL1A2 | NSN | NM_000089 | c.2123G>A (p.R708Q) |
| 1 | 1.3 | 7:150645534 | rs1805123 | het | KCNH2 | NSN | NM_000238 | c.2690A>C (p.K897T) |
| 1 | 1.3 | 8:19819536 | rs327 | homo | LPL | In | NM_000237 | c.1323-90T>G |
| 1 | 1.3 | 8:19819724 | rs328 | homo | LPL | Stop Gain | NM_000237 | c.1421C>G (p.S474X) |
| 1 | 1.3 | 10:72195439 | rs1904589 | het | NODAL | NSN | NM_018055 | c.494A>G (p.H165R) |
| 2 | 1.3 | 14:90863357 | rs12885713 | homo | CALM1 | UTR5 | NM_006888 | c.-218C>T |
| 2 | 1.3 | 5:172662024 | rs2277923 | het | NKX2-5 | SYN | NM_004387 | c.63A>G (p.E21E) |
| 2 | 1.3 | 10:72195439 | rs1904589 | het | NODAL | NSN | NM_018055 | c.494A>G (p.H165R) |
| 3 | 1.1* | 17:7123838 | rs28934585 | het | ACADVL | NSN | NM_000018 | c.194C>T (p.P65L) |
| 3 | 1.3 | 5:172662024 | rs2277923 | het | NKX2-5 | SYN | NM_004387 | c.63A>G (p.E21E) |
| 3 | 1.3 | 10:72195439 | rs1904589 | het | NODAL | NSN | NM_018055 | c.494A>G (p.H165R) |
| 3 | 1.3 | 1:237778084 | rs3766871 | het | RYR2 | NSN | NM_001035 | c.5656G>A (p.G1886S) |
| 4 | 1.1 | 10:88446830 | rs121908338 | het | LDB3 | NSN | NM_001080116 | c.349G>A (p.D117N) |
| 4 | 1.3 | 8:19819536 | rs327 | het | LPL | In | NM_000237 | c.1323-90T>G |
| 4 | 1.3 | 5:172662024 | rs2277923 | het | NKX2-5 | SYN | NM_004387 | c.63A>G (p.E21E) |
| 4 | 1.3 | 10:72195439 | rs1904589 | homo | NODAL | NSN | NM_018055 | c.494A>G (p.H165R) |
| 4 | 1.3 | 1:11906068 | rs5065 | het | NPPA | Stop Loss | NM_006172 | c.454T>C (p.X152R) |
| 4 | 1.2 | 19:49700017 | rs200038418 | het | TRPM4 | NSN | NM_017636 | c.2531G>A (p.G844D) |
| 5 | 1.3 | 14:90863357 | rs12885713 | homo | CALM1 | UTR5 | NM_006888 | c.-218C>T |
| 5 | 1.3 | 5:172662024 | rs2277923 | het | NKX2-5 | SYN | NM_004387 | c.63A>G (p.E21E) |
| 5 | 1.3 | 10:72195439 | rs1904589 | het | NODAL | NSN | NM_018055 | c.494A>G (p.H165R) |
| 5 | 1.3 | 1:11906068 | rs5065 | het | NPPA | Stop Loss | NM_006172 | c.454T>C (p.X152R) |
| 5 | 1.3 | 1:55518374 | rs148195424 | het | PCSK9 | NSN | NM_174936 | c.709C>T (p.R237W) |
| 5 | 1.3 | 3:38645420 | rs1805124 | homo | SCN5A | NSN | NM_198056 | c.1673A>G (p.H558R) |
| 6 | 1.2 | 12:22017410 | rs61688134 | het | ABCC9 | NSN | NM_005691 | c.2200G>A (p.V734I) |
| 6 | 1.3 | 1:76227022 | rs1061337 | het | ACADM | SYN | NM_001127328 | c.1173A>G (p.V391V) |
| 6 | 1.3 | 10:18828635 | rs58225473 | het | CACNB2 | NSN | NM_201596 | c.1965T>G (p.D655E) |
| 6 | 1.3 | 14:90863357 | rs12885713 | het | CALM1 | UTR5 | NM_006888 | c.-218C>T |
| 6 | 1.3 | 8:19819536 | rs327 | het | LPL | In | NM_000237 | c.1323-90T>G |
| 6 | 1.3* | 16:83941726 | rs2278037 | het | MLYCD | In | NM_012213 | c.642-5C>T |
| 6 | 1.3 | 5:172662024 | rs2277923 | het | NKX2-5 | SYN | NM_004387 | c.63A>G (p.E21E) |
| 6 | 1.3 | 10:72195439 | rs1904589 | homo | NODAL | NSN | NM_018055 | c.494A>G (p.H165R) |
| 6 | 1.3 | 1:11906068 | rs5065 | het | NPPA | Stop Loss | NM_006172 | c.454T>C (p.X152R) |
| 6 | 1.3 | 3:38645420 | rs1805124 | het | SCN5A | NSN | NM_198056 | c.1673A>G (p.H558R) |
| 6 | 1.2 | 19:49700017 | rs200038418 | het | TRPM4 | NSN | NM_017636 | c.2531G>A (p.G844D) |
| 7 | 1.2 | 2:21229160 | rs5742904 | het | APOB | NSN | NM_000384 | c.10580G>A (p.R3527Q) |
| 7 | 1.3 | 10:18828635 | rs58225473 | homo | CACNB2 | NSN | NM_201596 | c.1965T>G (p.D655E) |
| 7 | 1.3 | 8:19819536 | rs327 | homo | LPL | In | NM_000237 | c.1323-90T>G |
| 7 | 1.3 | 10:72195439 | rs1904589 | het | NODAL | NSN | NM_018055 | c.494A>G (p.H165R) |
| 7 | 1.2 | 3:38655278 | rs45620037 | het | SCN5A | NSN | NM_198056 | c.659C>T (p.T220I) |
| 8 | 1.3 | 4:114278277 | rs3733617 | het | ANK2 | NSN | NM_001148 | c.8503C>T (p.P2835S) |
| 8 | 1.3* | 16:83941726 | rs2278037 | het | MLYCD | In | NM_012213 | c.642-5C>T |
| 8 | 1.3 | 10:72195439 | rs1904589 | homo | NODAL | NSN | NM_018055 | c.494A>G (p.H165R) |
| 8 | 1.3 | 3:38645420 | rs1805124 | het | SCN5A | NSN | NM_198056 | c.1673A>G (p.H558R) |
| 8 | 1.2 | 1:26393843 | rs140523053 | het | TRIM63 | NSN | NM_032588 | c.143C>T (p.A48V) |
| 9 | 1.1* | 17:7123838 | rs28934585 | het | ACADVL | NSN | NM_000018 | c.194C>T (p.P65L) |
| 9 | 1.3 | 4:114278277 | rs3733617 | het | ANK2 | NSN | NM_001148 | c.8503C>T (p.P2835S) |
| 9 | 1.1* | 1:53679053 | rs1871748 | het | CPT2 | NSN | NM_000098 | c.1763C>G (p.S588C) |
| 9 | 1.3 | 8:19819536 | rs327 | het | LPL | In | NM_000237 | c.1323-90T>G |
| 9 | 1.3 | 5:172662024 | rs2277923 | homo | NKX2-5 | SYN | NM_004387 | c.63A>G (p.E21E) |
| 9 | 1.3 | 10:72195439 | rs1904589 | homo | NODAL | NSN | NM_018055 | c.494A>G (p.H165R) |
| 9 | 1.3 | 1:11906068 | rs5065 | het | NPPA | Stop Loss | NM_006172 | c.454T>C (p.X152R) |

*Variant classification in autosomal recessive disease; however, the patient in question is heterozygous for the variant.

^†^Cat (HGMD category after literature search): 1.1 = affects function in isolation; 1.2 = may or may not affect function in isolation; 1.3 = affects function as modifier

Underlined: arrhythmia associated gene

**chr** = chromosome; **del** = deletion; **FS** = frameshift; **GT** = genotype; **het** = heterozygous; **homo** = homozygous; **ID** = Pacemaker patient identification; **in** = intronic; **ins** = insertion; **NFS** = non-frameshift; **NSN** = nonsynonymous; **pos** = position; **SYN** = synonymous; **UTR5** = variant in 5’untranslated region

**Table F: Major co-morbidities at the time of pacemaker implantation in patients with ICCD or SSS without structural heart disease.**

| ­­­­­­­Co-morbidities⃰ | Number of patients |
| --- | --- |
| Hypertension | 4 |
| Alcohol abuse (current) | 2 |
| Hyperlipidemia | 2 |
| Chronic obstructive pulmonary disease | 1 |
| Seizures† | 1 |
| Hypothyroidism | 1 |
| Lacunar cerebrovascular accidents | 1 |

*Some patients had more than one co-morbidity present at the time of insertion

†Suspected due to history of alcohol abuse
